# A retrospective nationwide analysis of evolocumab use in Sweden and its effect on low-density lipoprotein cholesterol levels

**DOI:** 10.48101/ujms.v129.9618

**Published:** 2024-01-31

**Authors:** Maria K. Svensson, Stefan James, Annica Ravn-Fischer, Guillermo Villa, Lovisa Schalin, Thomas Cars, Stefan Gustafsson, Emil Hagström

**Affiliations:** a Department of Medical Sciences, Uppsala University, Uppsala, Sweden; b Uppsala Clinical Research Centre, Uppsala, Sweden; c Department of Cardiology, Sahlgrenska University Hospital, Gothenburg, Sweden; d Health Economics & Outcomes Research, Amgen (Europe) GmbH, Rotkreuz, Switzerland; e Medical Affairs, Amgen AB, Stockholm, Sweden; f Sence Research, Uppsala, Sweden

**Keywords:** Adherence, evolocumab, LDL-C, PCSK9 inhibitors, persistence, real-world evidence

## Abstract

**Background:**

Treatment with proprotein convertase subtilisin/kexin type 9 (PCSK9) inhibitors reduces low-density lipoprotein cholesterol (LDL-C) levels and decreases the incidence of major ischaemic events in clinical trials. However, less is known about the efficacy of PCSK9 inhibition in clinical practice. This study aimed to describe the change in LDL-C levels over time and LDL-C goal achievement in patients with/without atherosclerotic cardiovascular disease (ASCVD), who were prescribed evolocumab in clinical practice, and to describe adherence to and persistence with treatment.

**Methods:**

Patients in Sweden with at least one evolocumab prescription filled between July 2015 and May 2020 were included. Medical history and lipid-lowering therapy (LLT) were sourced from national registries. LDL-C levels before and after treatment initiation were assessed using medical records. Persistence with and adherence to evolocumab and oral LLT were assessed up to 12 months after treatment initiation using the refill-gap method and proportion of days covered, respectively.

**Results:**

Of the 2,360 patients with at least one prescription for evolocumab, 2,341 were included; 1,858 had ASCVD. Persistence with (76%) and adherence to (86%) evolocumab were high throughout the 12 months following initiation. Mean LDL-C levels decreased by 53% (95% confidence interval [CI]: 51–55%) in patients adherent to evolocumab (*n* = 567) and 59% (95% CI: 55–63%) in patients adherent to evolocumab and oral LLT (*n* = 186). Similar reductions in LDL-C were observed in patients with/without ASCVD. Reduced LDL-C levels remained stable during follow-up. Amongst patients adherent to evolocumab and those adherent to evolocumab and oral LLT, 23 and 55% achieved the LDL-C goal of <1.4 mmol/L, respectively.

**Conclusions:**

The evolocumab LDL-C-lowering effect observed in clinical trials was confirmed in clinical practice in Sweden, particularly in patients also treated with oral LLT. During follow-up, adherence to and persistence with evolocumab were high, with stable reduced levels of LDL-C during observation.

## Introduction

Evidence from numerous clinical and genetic studies has established that low-density lipoprotein cholesterol (LDL-C) causes atherosclerotic cardiovascular disease (ASCVD). There is a consistent dose-dependent association between the absolute magnitude of exposure of the vasculature to LDL-C and the risk of ASCVD; this effect appears to increase as the duration of exposure to LDL-C increases (1, 2). Furthermore, LDL-C reduction, in both primary and secondary prevention, translates to a lower rate of ASCVD events (3). Consequently, lowering LDL-C levels is one of the main therapeutic targets in reducing the risk of ASCVD (3).

Inhibition of proprotein convertase subtilisin/kexin type 9 (PCSK9) using monoclonal antibodies has been shown to lower LDL-C levels by approximately 60% and to reduce the risk of major ASCVD events across a wide range of different patient populations in clinical trials, when used in monotherapy or in addition to other lipid-lowering therapy (LLT) (4–7). Considering the beneficial effects of PCSK9 inhibition alongside potential adverse effects of oral LLT, particularly statin-associated muscle symptoms (8), PCSK9 inhibitors offer an attractive treatment strategy.

Whilst the efficacy of PCSK9 inhibition has been established in several randomised clinical trials, there is no comprehensive evidence from public health settings (9, 10); therefore, additional real-world data are required to describe the effectiveness of PCSK9 inhibition in routine clinical practice. Additionally, little is known about how oral LLT usage patterns change after the initiation of PCSK9 inhibition when the two are used in combination therapy. Therefore, we assessed the change in LDL-C levels and LDL-C goal achievement before and after the initiation of evolocumab treatment, in a population- and registry-based nationwide cohort of patients prescribed the PCSK9 inhibitor evolocumab.

## Methods

### Patients and data sources

This study covered data for all residents in Sweden, all of whom have universal access to healthcare with a negligible co-payment for healthcare visits, hospitalisations and medications (11). Data were sourced from the Swedish Prescribed Drug Register and the National Patient Register at the National Board of Health and Welfare in Sweden. The Swedish Prescribed Drug Register contains information describing medications dispensed to each Swedish resident, logged according to the Anatomical Therapeutic Chemical (ATC) classification system (12). The National Patient Register collects information regarding inpatient and outpatient care, as well as any use of other specialist clinics in ambulatory care. Diagnoses and procedures are coded according to the International Classification of Diseases (ICD) (13), the Nordic Medico-Statistical Committee Classification of Surgical Procedures system (14) and the National Classification System of Clinical Procedure (15). Data from the National Patient Register were available for 1997–2019. Lipid levels were retrieved from the electronic medical records (EMRs) of five regions in Sweden (Stockholm [2005–2020], Dalarna [2002–2020], Uppsala [2005–2020], Skåne [2011–2020] and Västra Götaland [2000–2020]), covering more than half of the population of Sweden. The study protocol was approved by the Swedish Ethical Review Authority (approval number 2019-04586).

### Study cohort

An overall nationwide cohort was formed of all residents of Sweden who had at least one prescription for evolocumab (ATC code: C10AX13) filled between 1 July 2015 and 31 May 2020. Patients were excluded if they had recorded lipoprotein apheresis treatment (national classification code: DR001) or had more than two LDL-C measurements conducted in a single day with a significant reduction in LDL-C level between measurements, potentially indicating treatment for lipoprotein apheresis. The 12-digit personal identity number (16), unique to all Swedish residents, was used to link this cohort to the available data (e.g. patient demographics, diagnoses of comorbid conditions including familial hypercholesterolaemia [FH], healthcare use, treatment with other LLT [such as statins and/or ezetimibe] and lipid profiles). Data from all patients eligible for inclusion in this study were combined for the analyses, regardless of when treatment was initiated. Of note, the national criteria for evolocumab reimbursement were amended in 2019, lowering the LDL-C threshold for reimbursement from ≥4.0 to ≥2.5 mmol/L in ASCVD patients on a maximally tolerated dose of LLT (statins and/or ezetimibe) and setting the threshold to ≥3.0 mmol/L for patients with heterozygous FH without ASCVD, as well as the inclusion of patients with homozygous FH (17). Furthermore, it is recommended that treatment with PCSK9 inhibitors is initiated by a physician who specialises in cardiology, endocrinology or internal medicine (18).

### Characterisation of patients treated with evolocumab

Patient characteristics collected were ASCVD diagnoses (coronary heart disease, coronary revascularisation, stroke, peripheral revascularisation and peripheral artery disease, myocardial infarction and cerebral infarction), other diseases of the circulatory system, diabetes mellitus, chronic kidney disease, lipoprotein metabolism disorders or other dyslipidaemias, treatment with lipoprotein apheresis and a diagnosis of FH (Supplementary Methods 1). Information on the use of background LLT (statins and/or ezetimibe), in addition to evolocumab, was also collected and is presented in Table 1.

**Table 1:** Characteristics of the overall study population.

	Overall	With ASCVD	Without ASCVD
N	2,341	1,858	483
Female, n (%)	1,015 (43.4)	727 (39.1)	288 (59.6)
Age (years), mean (SD)	63.4 (11.1)	65.2 (9.8)	56.6 (12.9)
**Medical history, n (%)**			
Coronary heart disease	1,747 (74.6)	1,747 (94.0)	0 (0)
Acute myocardial infarction	1,135 (48.5)	1,135 (61.1)	0 (0)
All stroke	285 (12.2)	285 (15.3)	0 (0)
Peripheral artery disease	251 (10.7)	251 (13.5)	0 (0)
Diabetes mellitus (type 1 and 2)	492 (21.0)	438 (23.6)	54 (11.2)
Background LLT,^a^ n (%)	1,495 (63.9)	1,231 (66.3)	264 (54.7)
Ezetimibe and any statin	600 (25.6)	493 (26.5)	107 (22.2)
Ezetimibe monotherapy	403 (17.2)	345 (18.6)	58 (12.0)
High-intensity statin	274 (11.7)	221 (11.9)	53 (11.0)
Moderate-intensity statin	194 (8.3)	155 (8.3)	39 (8.1)
Low-intensity statin	24 (1.0)	17 (0.9)	7 (1.4)
**LDL-C**			
Baseline measure available, n (%)	1,025 (43.8)	808 (43.5)	217 (44.9)
Baseline value (mmol/L),^b^ mean (SD)	4.3 (1.7)	4.0 (1.4)	5.3 (2.0)
**FH, n (%)**			
FH (ICD-10 E78.0A)	230 (9.8)	134 (7.2)	96 (19.9)
Phenotypical FH			
Definite FH, DLCN score >8	24 (1.0)	*	*
Probable FH, DLCN score 6–8	111 (4.7)	83 (4.5)	28 (5.8)
Possible FH, DLCN score 3–5	645 (27.6)	497 (26.7)	148 (30.6)
Unlikely FH, DLCN score below 3	431 (18.4)	386 (20.8)	45 (9.3)
DLCN score missing	900 (38.4)	735 (39.6)	165 (34.2)

a Filled prescriptions for other LLT where the number of days covered (including a 25% grace period) overlaps the date evolocumab treatment was initiated.

b The most recent measurement of LDL-C recorded within the 3 months prior to the date evolocumab treatment was initiated.

* Not reported due to less than five patients in cohort.

ASCVD: atherosclerotic cardiovascular disease; DLCN: Dutch Lipid Clinic Network; FH: familial hypercholesterolaemia; ICD: International Classification of Diseases; LDL-C: low-density lipoprotein cholesterol; LLT: lipid-lowering therapy; SD: standard deviation.

After approved confidentiality assessment, information was extracted from the EMR databases and linked and pseudonymised. The data extraction mainly includes information recorded by public healthcare providers. EMRs of five regions in Sweden were used to determine lipid levels from recorded laboratory values, which included LDL-C (using the most recent measurement of LDL-C within the 3 months before the first prescription of evolocumab was filled), high-density lipoprotein cholesterol, total cholesterol and triglycerides. If measurements were not available, LDL-C was estimated using the Friedewald equation in patients with triglycerides <4.5 mmol/L (19).

Patients were identified as having FH using the associated ICD-10 code; however, the ICD-10 code was only implemented into the Swedish ICD system on 1 January 2019. Therefore, the highest LDL-C or total cholesterol concentration on record, together with the presence of premature cardiovascular disease according to type, age and sex, was used to identify potential patients with phenotypical FH, according to the Dutch Lipid Clinic Network (DLCN) criteria (Supplementary Methods 2) (20), which were applied where data were available. Using the DLCN criteria, patients with ≥6 points were considered to have phenotypic FH, whereas patients with ≤5 points were considered not to have FH.

### Persistence with and adherence to evolocumab

Persistence is the duration of time from prescription to evolocumab discontinuation and was assessed using the refill-gap method (21–23). The number of days covered by a prescription (i.e. the number of days until an evolocumab supply was expected to end) was calculated by multiplying the total number of evolocumab syringes purchased when that prescription was filled by the prescribed dose (mg). To acknowledge that prescription patterns and medication use are not always consistent, a permissible last gap of 56 days was added to the number of days covered by a prescription for the base case analysis, to allow for expected variations in adherence. A 56-day gap has been used previously to define persistence in the analysis of persistence with evolocumab in a Canadian population (24). If the next prescription was not filled before this total period of coverage ended, the patients were marked as non-persistent for that period.

Adherence is the extent to which the prescribed interval and dose of evolocumab treatment were followed (21) and was assessed using the proportion of days covered (25):


$$\text{Proportion of day scovered}=100\times \frac{\text{Number of days covered}}{\text{Total number of days}}$$

where the number of days covered in a given period was calculated by multiplying the total number of evolocumab syringes purchased (across one or more prescriptions) during that period by the prescribed dose (mg).

If the prescribed dose was missing, a standard dose of one prefilled syringe every 2 weeks was imputed. In addition to presenting unadjusted values, adherence was adjusted for persistence by excluding patients who were likely to have discontinued their treatment (those who had not filled another prescription within the coverage period, including a 56-day permissible gap). Those who had a proportion of days covered of ≥0.80 were considered adherent (23, 26). Persistence with and adherence to evolocumab were assessed at 6, 9, 12, 24 and 36 months from the date the evolocumab treatment was initiated (each month was standardised to a duration of 30 days).

Sensitivity analyses were conducted for the assessment of persistence with (Supplementary Methods 3) and adherence to evolocumab, which address other reasons for inconsistencies in medication use. Sensitivity analyses included allowing a 28-day permissible gap, allowing a 25% grace period, accounting for days spent in inpatient care for a cardiovascular disease admission and accounting for prescription overlap. Sensitivity analyses also distinguished between first and last incidents of non-persistence.

For the base case and sensitivity analyses, a last incident of non-persistence was defined as cases where there was no subsequent filled prescription for evolocumab. Patients were censored when deemed non-persistent or at death. To be included in these analyses, the follow-up period for a given patient needed to be at least 28 or 56 days, dependent on the analysis being conducted.

Analyses were performed on data from the overall cohort and stratified by history of clinical ASCVD at the time the evolocumab treatment was first initiated.

### Persistence with and adherence to background LLT

Persistence with and adherence to background LLT were assessed if other LLT (statins and/or ezetimibe) were used at the time a patient initiated evolocumab treatment. The assessment was performed using the same methods detailed earlier, and data for persistence with and adherence to other LLT were presented as for the evolocumab analyses. Adherence data were also considered when assessing changes in LDL-C over time following evolocumab treatment.

### Changes in LDL-C following initiation of evolocumab treatment

Patients from five regions of Sweden where LDL-C data were available (Stockholm, Dalarna, Uppsala, Skåne and Västra Götaland) were included in the analyses if they had at least one LDL-C measurement within the 180 days prior to evolocumab treatment initiation (pre-evolocumab treatment level), and at least one other LDL-C measurement within the 180 days after evolocumab treatment initiation (post-evolocumab treatment level). An assumption was made that evolocumab treatment began after the first evolocumab prescription was filled (27, 28).

Changes in absolute LDL-C (mmol/L) levels over time and the mean relative reduction (%) from pre-evolocumab treatment levels are presented for the overall cohort and for those with (secondary prevention) and without ASCVD (primary prevention) at the time evolocumab treatment was first initiated. For each group of interest, changes in LDL-C were also assessed in 1) all patients in each cohort, 2) in patients adherent to evolocumab (whose proportion of days covered, unadjusted for persistence, with evolocumab was ≥80% during the first 180 days from the date treatment was initiated) and 3) in patients adherent to evolocumab and LLT (whose proportion of days covered with other LLT was ≥80% for the 90 days before and the 90 days after evolocumab treatment was initiated, in addition to a proportion of days covered ≥80% for evolocumab). The estimated LDL-C level 1 day before evolocumab treatment was initiated was compared with the estimated level 90 days after initiation.

LDL-C goal achievement at 90 days was assessed according to the recommendation in the 2019 and 2016 European Society of Cardiology/European Atherosclerosis Society (ESC/EAS) guidelines. Current recommended LDL-C goals of <1.8 mmol/L for high-risk patients (including those with FH) and <1.4 mmol/L for very-high-risk patients (including patients with ASCVD and patients with FH and additional risk factors) (8) were revised from previously recommended LDL-C goals of <2.6 mmol/L for high-risk patients and <1.8 mmol/L for very high-risk patients, which were current at the time data were collected (29).

### Statistical analysis

Patient baseline characteristics were presented descriptively. Data were summarised by persistence with and adherence to evolocumab and by achieved mean LDL-C levels, defined as the last on-treatment LDL-C level recorded at 90 days after evolocumab was initiated. Regarding adherence, the proportion of days covered was reported as the mean (standard deviation [SD]) and median (interquartile range [IQR]). Regarding persistence, the number of days from the date that evolocumab treatment was first initiated to the first and last incidents of non-persistence is presented using Kaplan–Meier curves, together with the proportion of patients considered persistent with the prescribed evolocumab treatment.

The patients included in these analyses had a varying number of LDL-C measurements within the time period of interest, and these measurements may have been performed at unequally spaced timepoints. A generalised least squares regression model, assuming a first-order autoregressive error for serially correlated data, was therefore used in the analyses (27, 28). LDL-C point estimates with 95% confidence intervals (CIs) were presented. CIs for the percent reduction were calculated using 10,000 bootstrap samples. The CIs of the percent reduction were calculated using the bias-corrected and accelerated (BCa) bootstrap method as implemented in the R boot package.

Regarding LDL-C goal achievement, a multi-state Markov model of panel data (30) was fitted with the transient LDL-C states: ‘<1.4 mmol/L’, ‘≥1.4 to <1.8 mmol/L’ and ‘≥1.8 mmol/L’. This model is described in detail in Supplementary Methods 4.

## Results

A total of 2,360 patients in Sweden had at least one prescription for evolocumab filled during the study period. Of these, 2,341 were included in this study, with 19 patients excluded due to reported or potentially indicated lipoprotein apheresis treatment. A total of 1,858 (79%) patients had a prior ASCVD diagnosis (Table 1). Of the patients without a prior ASCVD diagnosis (483 [21%]), 20% had an ICD-10 diagnosis of FH and 6% had phenotypical FH. In those with ASCVD, 7% had an ICD-10 diagnosis of FH and 5% had phenotypical FH.

Amongst those with a history of ASCVD, 94% previously had a coronary event (e.g. myocardial infarction, unstable angina, percutaneous coronary intervention and coronary artery bypass graft) and 24% had diabetes mellitus of any type. Overall, 64% of patients were undergoing treatment with an oral LLT, with a lower proportion (55%) in patients without prior ASCVD. The mean pre-evolocumab treatment LDL-C level was 4.0 mmol/L (SD: 1.4) in patients with prior ASCVD, and 5.3 mmol/L (SD: 2.0) in those without ASCVD at the initiation of evolocumab treatment.

The median follow-up period from the date evolocumab treatment was first initiated to the end of study was 376 days (IQR: 184–690). The median number of days covered by a dispensation of evolocumab was 84, corresponding to six injections with a dose of one injection every 2 weeks.

### Persistence with and adherence to evolocumab and other LLT

**Figure 1:**
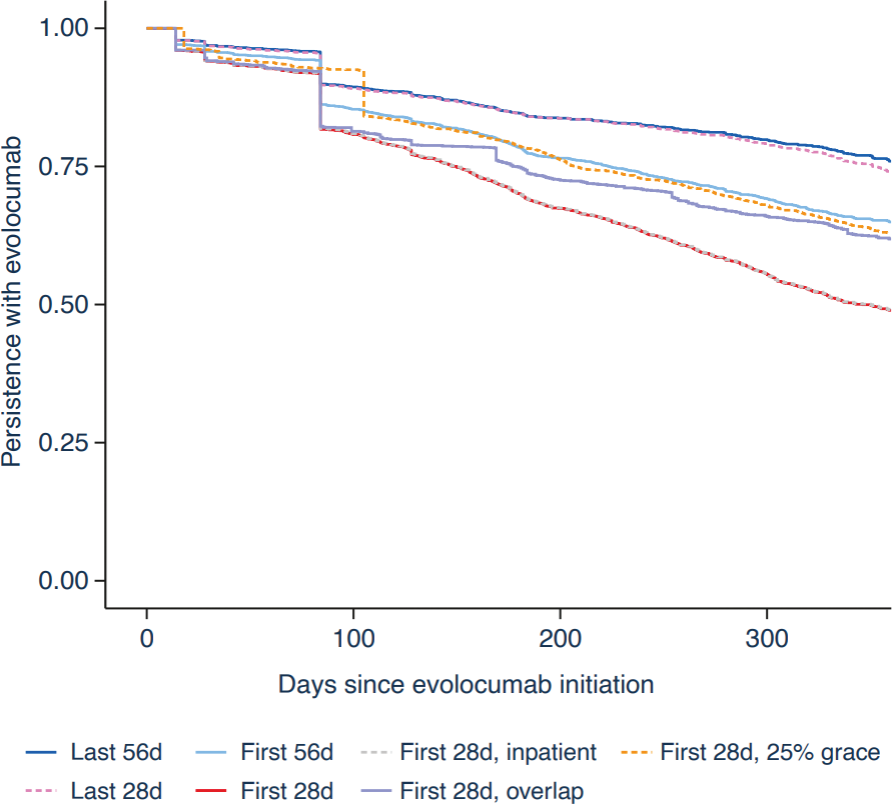
Kaplan–Meier curve of persistence with evolocumab in the overall cohort during the first 12 months of follow-up. The seven lines represent the different gap definitions used in the analyses: First 56d and Last 56d: base case where a permissible gap of 56 days was added to the number of days covered; First 28d and Last 28d: sensitivity analysis where a permissible gap of 28 days was added to the number of days covered; First 28d, inpatient: sensitivity analysis where the number of days a patient spent in inpatient care for a cardiovascular disease admission was added (in addition to a permissible gap of 28 days) to the number of days covered; First 28d, overlap: sensitivity analysis where the number of overlapping days between a new evolocumab prescription being filled before the end of the previous coverage period was added (in addition to a permissible gap of 28 days) to the subsequent coverage period; First 28d, 25% grace: sensitivity analysis where coverage periods, inclusive of a permissible gap of 28 days, were extended by an additional 25%.

The proportion of patients persistent with evolocumab was 76% over the first 12 months of treatment for the overall cohort when considering the last permissible 56-day gap. A substantial reduction in persistence is observable at Day 84 in all analyses presented due to some patients discontinuing evolocumab treatment after their first filled prescription (Figure 1). Sensitivity analysis showed that 74% of patients in the overall cohort were persistent with evolocumab when considering a last permissible 28-day gap (Supplementary Results 1 – Supplementary Table 1). A similar proportion of patients were shown to be persistent in those with (76%) or without (77%) a prior diagnosis of ASCVD (Supplementary Results 2 – Supplementary Figures 1 and 2). Persistence declined across the entire cohort in the second and third years following evolocumab treatment initiation (Supplementary Results 1 – Supplementary Table 1). In the overall cohort, persistence with other LLT during the same 12-month period was relatively poor compared with persistence with evolocumab, with 62% (last permissible 56-day gap) of patients considered persistent in the base analysis (Supplementary Results 3 – Supplementary Figure 3).

**Figure 2:**
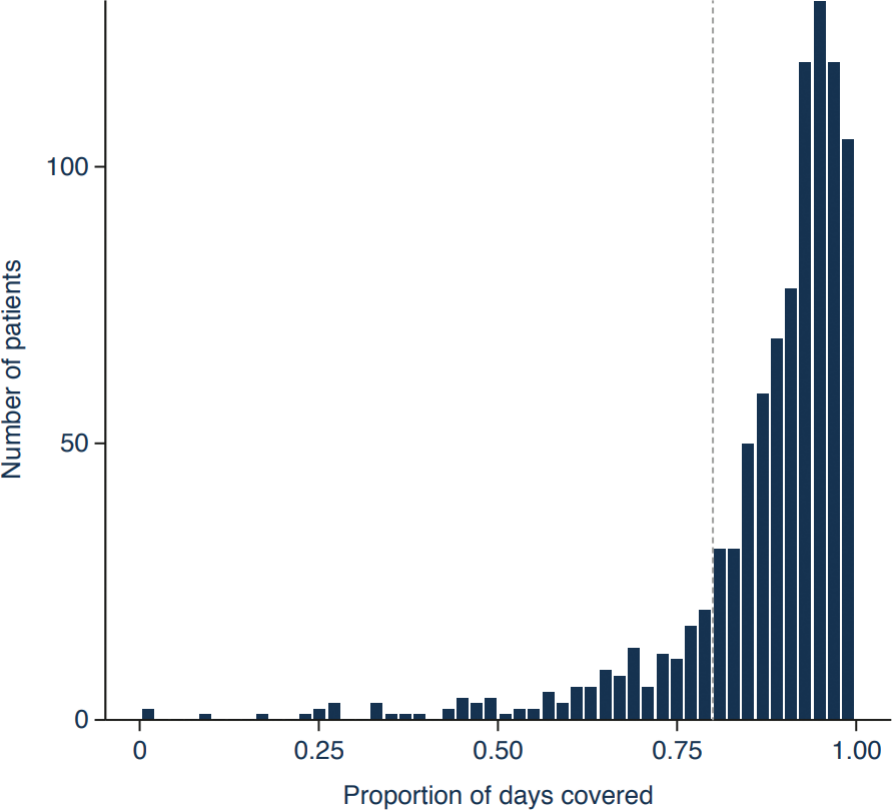
Adherence to evolocumab in the overall cohort during the first 12 months of follow-up. The dashed line indicates a proportion of days covered of 0.8.

The median proportion of days covered in patients who had at least 12 months of follow-up (*n* = 941) was 93% (IQR: 86–97). Of those patients who had at least 12 months of follow-up and were considered persistent with treatment, 86% were adherent to evolocumab (proportion of days covered ≥0.80) (Figure 2). There was little variation in adherence between those with (86%) and without (83%) ASCVD; and adherence improved slightly with the extended allowances for inconsistencies in prescription patterns and medication usage (Supplementary Results 4 – Supplementary Tables 2–4). A slight improvement in adherence was reported when a 25% grace period was considered and when prescription overlap was accounted for, showing that 92 and 91% of patients were adherent to evolocumab, respectively (Supplementary Results 4 – Supplementary Table 2). Adherence was still high in the second (81%) and third (79%) years after evolocumab treatment was initiated in those who remained persistent with treatment throughout that period (Supplementary Results 4 – Supplementary Table 2).

### Changes in LDL-C levels following evolocumab treatment

**Figure 3:**
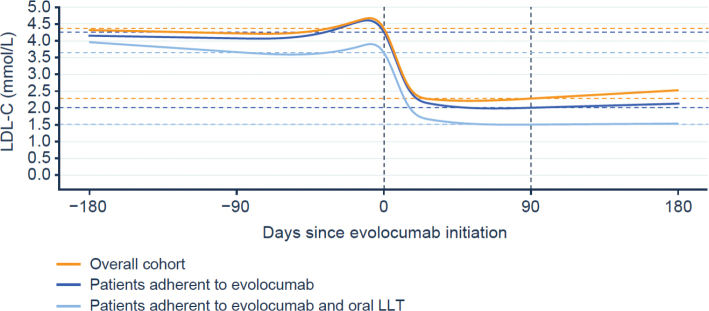
Change in LDL-C level prior to evolocumab treatment initiation to 90 days after treatment in the overall cohort, patients adherent to evolocumab, and patients adherent to evolocumab and oral LLT. Data are based patients from the overall cohort (*n* = 724); patients who were adherent to evolocumab treatment (*n* = 567); and patients who were adherent to evolocumab treatment and oral LLT (*n* = 186). All patients included had recorded measurements of LDL-C levels during the 180 days before and the 180 days after evolocumab treatment was initiated. The upper horizontal dashed line denotes the pre-evolocumab treatment mean LDL-C level, and the lower dashed line denotes the post-evolocumab treatment mean LDL-C level, coloured separately for each analysis group. The baseline LDL-C values are different for each analysis group. The dashed vertical lines represent the day before treatment was initiated at day 0, and the post-evolocumab treatment measurement 90 days after initiation. LDL-C: low-density lipoprotein cholesterol; LLT: lipid-lowering therapy.

**Figure 4:**
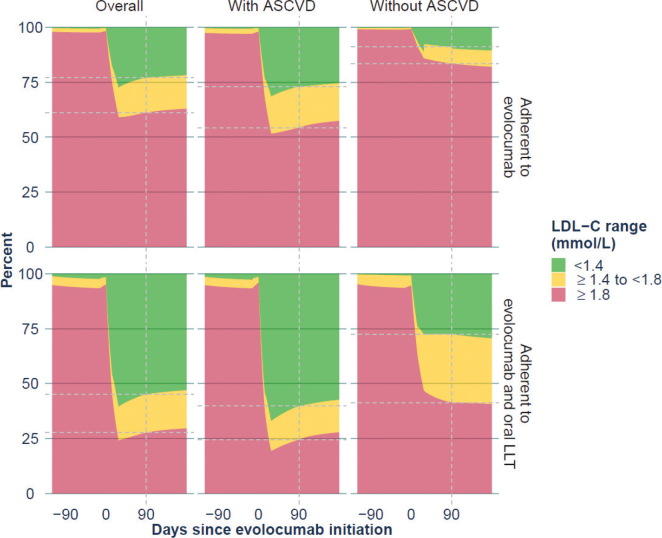
Proportion of patients in LDL-C categories over time since evolocumab initiation in the overall cohort, patients adherent to evolocumab and patients adherent to evolocumab and oral LLT, with and without ASCVD. ASCVD: atherosclerotic cardiovascular disease; LDL-C: low-density lipoprotein cholesterol; LLT: lipid-lowering therapy.

From the overall cohort, 724 patients had recorded measurements of LDL-C available within the 180 days before and the 180 days after evolocumab treatment was first initiated (Table 2). Amongst these patients, there was a 48% (95% CI: 46–50%) decrease in mean LDL-C levels 90 days after evolocumab treatment was initiated (Supplementary Results 5 – Supplementary Figure 4). For those who were considered adherent to evolocumab during the first 180 days of their treatment, mean LDL-C levels decreased by 53% (95% CI: 51–55%) after 90 days (Supplementary Results 5 – Supplementary Figure 5). Mean LDL-C levels observed in those who were adherent to evolocumab and also to oral LLT decreased by 59% (95% CI: 55–63%) after 90 days (Supplementary Results 5 – Supplementary Figure 6). The change in LDL-C level at 90 days following evolocumab initiation in these three groups (the overall cohort, those patients adherent to evolocumab and those patients adherent to evolocumab and oral LLT) is illustrated in Figure 3. Similar reductions in mean LDL-C levels were found in those with (overall cohort: 50%; those adherent to evolocumab: 55%; those adherent to evolocumab and oral LLT: 58%) and without ASCVD, the majority of whom potentially had FH (overall cohort: 41%; those adherent to evolocumab: 47%; those adherent to evolocumab and oral LLT were omitted due to a low number of patients in this subgroup [data not shown]). In all cases, the reduction in mean LDL-C levels remained stable up to 6 months (Supplementary Results 5 – Supplementary Figures 7–11). Based on model estimates, in the overall cohort, the LDL-C goal of <1.8 mmol/L was achieved by 39 and 73% of patients adherent to evolocumab and to evolocumab and oral LLT, respectively, whilst 23 and 55% achieved the LDL-C goal of <1.4 mmol/L. Amongst patients with ASCVD, the LDL-C goal of <1.8 mmol/L was achieved by 46 and 75% of patients adherent to evolocumab and to evolocumab and oral LLT, respectively, whilst 27 and 60% achieved the LDL-C goal of <1.4 mmol/L. Amongst patients without ASCVD, the LDL-C goal of <1.8 mmol/L was achieved by 17 and 59% of patients adherent to evolocumab and to evolocumab and oral LLT, respectively, whilst 9 and 28% achieved the LDL-C goal of <1.4 mmol/L (Figure 4). The difference in LDL-C goal achievement between patients with ASCVD and patients without ASCVD can be explained by the higher reimbursement LDL-C threshold in the latter group.

**Table 2:** Changes in LDL-C levels prior to evolocumab treatment initiation to 90 days after treatment.

	n	Pre-evolocumab treatment LDL-C mean, mmol/L	Post-evolocumab treatment LDL-C mean, mmol/L	Absolute change, mmol/L (95% CI)	Mean relative change, % (95% CI)
**Overall cohort**	724	4.4	2.3	–2.1 (–2.3, –1.9)	–48 (–50, –46)
Adherent to evolocumab^a^	567	4.3	2.1	–2.2 (–2.4, –2.1)	–53 (–55, –51)
Adherent to evolocumab and LLT^b^	186	3.6	1.5	–2.1 (–2.5, –1.8)	–59 (–63, –55)
**Cohort with ASCVD**	571	4.1	2.0	–2.1 (–2.3, –1.9)	–50 (–53, –48)
Adherent to evolocumab^a^	447	4.0	1.8	–2.2 (–2.4, –2.0)	–55 (–57, –52)
Adherent to evolocumab and LLT^b^	152	3.5	1.5	–2.0 (–2.4, –1.7)	–58 (–63, –53)
**Cohort without ASCVD^c^**	153	5.2	3.0	–2.2 (–2.6, –1.7)	–41 (–46, –36)
Adherent to evolocumab^a^	120	5.2	2.7	–2.5 (–2.9, –2.0)	–47 (–52, –42)

a Proportion of days covered (not adjusted for persistence) with evolocumab ≥0.8 at 6 months after the date evolocumab treatment was initiated.

b Proportion of days covered (not adjusted for persistence) with evolocumab ≥0.8 at 6 months after the date evolocumab treatment was initiated and the proportion of days covered with other LLT ≥0.8 at 3 months before and 3 months after the date evolocumab treatment was initiated.

c The cohort of patients without ASCVD who were adherent to evolocumab and LLT constituted a small subgroup and, therefore, were omitted from analysis.

ASCVD: atherosclerotic cardiovascular disease; CI: confidence interval; LDL-C: low-density lipoprotein cholesterol; LLT: lipid-lowering therapy.

## Discussion

In this nationwide study of patients who were prescribed evolocumab in Sweden, national registry data for comorbidities, demographics and prescribed drugs were combined with information from the EMR covering approximately 60% of the Swedish population. The three key findings were as follows: 1) mean LDL-C was reduced by 53% in patients who were adherent to evolocumab, when not concurrently adherent to oral LLT, and 59% if patients were adherent to evolocumab and oral LLT; 2) mean LDL-C reduction was stable over time following evolocumab initiation; and 3) adherence and persistence to evolocumab remained high during the 3 years of observation.

Although there is limited real-world evidence compared with that available from clinical trials, several studies have assessed the usage patterns and efficacy of PCSK9 inhibitors in routine clinical practice. Whilst the majority of this research provides little insight due to unrepresentative samples, the very few studies that provide data representative of a broader region or wider nation are still limited by either small sample sizes or relatively short follow-up (31–33). The recently published HEYMANS study provides the most comprehensive analysis to date, assessing the efficacy of evolocumab according to reimbursement criteria in over 1,900 patients from clinical practice centres across 12 European countries (10). The nationwide study presented here adds to the HEYMANS study and the overall body of research, by assessing both usage patterns and the efficacy of evolocumab with complete coverage, well validated health registries and unique data on LDL-C levels for more than half the entire Swedish population.

Similar to data from observational studies that evaluated PCSK9 inhibition, patients who were prescribed evolocumab in Sweden had pre-treatment baseline LDL-C levels far from treatment goals, despite being at high-risk of suffering a cardiovascular event. In the HEYMANS study, disparities between local reimbursement criteria and clinical guidelines were suggested to contribute to the large gap between actual patient LDL-C levels and treatment goals (10). In the HEYMANS study, patients initiating evolocumab treatment had baseline LDL-C levels three times higher than the recommended threshold for initiating treatment with PCSK9 inhibitors (10). The high baseline LDL-C levels measured in the Swedish cohort cannot solely be explained by narrow reimbursement criteria. However, the findings from the HEYMANS study echo other recent observational data from patients with ASCVD who also had LDL-C levels far from prevention goals (34–36). The reimbursement criteria for evolocumab shifted during the study, but for the majority of the patients included in this study, it was mandated that evolocumab could only be prescribed to patients already prescribed the maximally tolerated dose of LLT (statins and/or ezetimibe), and if there was a diagnosis of ASCVD with LDL-C of ≥2.5 mmol/L, or if the patient had heterozygous FH, without ASCVD and with LDL-C of ≥3.0 mmol/L (17). Patients with homozygous FH could also receive evolocumab (17).

Most of the patients included in this study had been previously treated with both statins and ezetimibe; however, approximately one-third were not using oral LLT at the time evolocumab treatment was initiated, potentially due to statin intolerance. This finding has also been described in the Pan European EUROASPIRE (European Action on Secondary and Primary Prevention by Intervention to Reduce Events) surveys of patients with coronary artery disease and FH (36, 37). Persistence with oral LLT declined in the 12 months after evolocumab treatment was initiated. Declining adherence and persistence to oral LLT over time translates to both higher LDL-C levels and a worse prognosis (38, 39). In this study, we have no insight into the reasons for lower adherence and persistence to LLT, such as the presence of statin-associated muscle symptoms; however, this has been the case in several other observational cohorts. It could also be speculated that discontinuation of oral LLT may be caused by treatment goal achievement.

Persistence with and adherence to evolocumab over the first 12 months of treatment were high, which is consistent with the results of the HEYMANS study (10). High adherence to and persistence with evolocumab were also reported in the AT-TARGET-IT observational study (40). Importantly, the LDL-C lowering following evolocumab treatment initiation in this study was similar to the reduction observed in the FOURIER (Further Cardiovascular Outcomes Research with PCSK9 Inhibition in Subjects with Elevated Risk) trial and that seen, following alirocumab treatment, in the ODYSSEY OUTCOMES trial (4–6). Consistent with the HEYMANS study (10), the largest reductions in LDL-C levels were observed in individuals adherent to both evolocumab and oral LLT, further supporting the importance of combination therapy as standard of care (41). The mean 59% LDL-C reduction reported for patients adherent to evolocumab and oral LLT in our study translated to 55% of patients achieving LDL-C goals of <1.4 mmol/L. This was similar to the HEYMANS study, which reported a 58% median LDL-C reduction, translating to 58% of patients achieving the LDL-C goal (10). Data from the AT-TARGET-IT study further support these results, reporting a 65% median LDL-C reduction and translating to 64% of patients achieving the LDL-C goal (40). The reduction in cardiovascular disease risk is directly related to the lowering of LDL-C (8). Additionally, it has been shown that earlier initiation of statin therapy in those with acute coronary syndrome is favourable, compared with delayed initiation (42). There are less data available on the timing of PCSK9 inhibitor treatment initiation after an ASCVD event; however, data from a sub-study of the FOURIER trial showed improved prognosis if evolocumab treatment was initiated within 1 year after an myocardial infarction, as compared with a delayed initiation of therapy (43). Further research is needed to determine the optimal timing of initiating intensive LLT beyond oral LLT in relation to an acute cardiovascular event or coronary revascularisations. The interactions between statins, PCSK9 inhibitors, LDL-C and cardiovascular risk are multifaceted and are influenced by genetic, lifestyle and environmental factors. Exploratory data suggest that the combination of statins and PCSK9 inhibition may have an additive effect (44). Interestingly, a recent observational study reported that men treated with PCSK9 inhibitors showed larger LDL-C reductions than women (45). This highlights the importance for future research on real-world use of PSCK9 inhibitors in different patient groups.

There are limitations to this study. This study provided insights on the real-world use of evolocumab in Sweden and was conducted by the manufacturer at the request of the Dental and Pharmaceutical Benefits Agency (TLV), as part of the price and reimbursement conditions agreed for evolocumab in January 2019. The study, therefore, did not consider alirocumab, the other PCSK9 inhibitor also reimbursed in Sweden at the time data were collected. The large disparity between the elevated LDL-C levels pre-evolocumab treatment in this cohort and the guidelines for initiating PCSK9 inhibition can be partly attributed to selection bias. Indeed, patients were enrolled into the study based on their initiation of evolocumab treatment, and evolocumab treatment was initiated due to elevated LDL-C and under reimbursement criteria. Phenotypic FH was defined utilising the DLCN criteria, where data were available, based on treated or untreated LDL-C levels and the presence of premature cardiovascular disease, with no information about family history for cardiovascular disease/hypercholesterolaemia or clinical signs of FH. Furthermore, LDL-C levels for DLCN scoring were not corrected for any LLT. The proportion of individuals with FH is likely underestimated, in the absence of this information or corrections. The duration of time between the pre-evolocumab treatment LDL-C measurement and the initiation of evolocumab treatment varied greatly between patients included in the study. In addition, the duration of exposure to evolocumab between LDL-C assessments also varied significantly between each patient. However, this was taken into account with the statistical methods utilised, revealing a strong trend in the reduction of LDL-C levels after the initiation of evolocumab treatment. There is no information detailing whether patients who filled their evolocumab prescription started therapy. Additionally, there was a lack of information explaining why (e.g. adverse effects) persistence and adherence with oral LLT declined. The use of retrospective data requires making assumptions with regard to prescription patterns and medication use, which may differ from real-world use. Thus, adherence to and persistence with treatment may have been affected by inconsistencies in medication use. However, sensitivity analyses on adherence and persistence generally supported the base case analysis (which considered a 56-day permissible gap). A high proportion of patients without a history of ASCVD had limited data in the health registries due to low utilisation of healthcare, observations based on those data should be treated with caution.

In conclusion, in this nationwide study of all patients prescribed evolocumab in Sweden, the LDL-C-lowering effects observed in randomised clinical trials were also observed in clinical practice. High adherence to evolocumab, especially if evolocumab was prescribed in combination with oral LLT, might be associated with the greatest reductions in LDL-C. However, no formal comparisons were performed in this study and may be warranted in future research.

## Data Availability

The data underlying this article cannot be shared publicly due to the privacy of individuals who participated in this study. The data will be shared on reasonable request to the corresponding author.

## OCRID IDs:

Maria K Svensson https://orcid.org/0000-0001-7545-5585

Stefan James https://orcid.org/0000-0003-4413-9736

Annica Ravn-Fischer https://orcid.org/0000-0003-2585-1592

Guillermo Villa https://orcid.org/0000-0003-4484-8858

Lovisa Schalin https://orcid.org/0009-0000-3541-6514

Thomas Cars https://orcid.org/0009-0008-0912-8226

Stefan Gustafsson https://orcid.org/0000-0001-5894-0351

Emil Hagstrӧm https://orcid.org/0000-0003-3221-0144
