# Supplementary material for: A retrospective nationwide analysis of evolocumab use in Sweden and its effect on low-density lipoprotein cholesterol levels
Source: Ups J Med Sci. 2024 Jan 31;129:10.48101/ujms.v129.9618. doi: 10.48101/ujms.v129.9618 (PMC10845886; doi:10.48101/ujms.v129.9618)
Supplement: Supplementary file 1 [file UJMS-129-9618-s001.pdf]

SUPPLEMENTARY INFORMATION

A retrospective nationwide analysis of evolocumab use in Sweden and its effect on low-density lipoprotein cholesterol levels

Maria K. Svensson[a], Stefan James[b,c], Annica Ravn-Fischer[d], Guillermo Villa[e], Lovisa Schalin[f], Thomas Cars[g], Stefan Gustafsson[g], Emil Hagström[b,c]

[a]Department of Medical Sciences, Uppsala University and Uppsala Academic Hospital, Uppsala, Sweden; [b]Department of Medical Sciences, Uppsala University, Uppsala, Sweden; [c]Uppsala Clinical Research Centre, Uppsala University, Uppsala, Sweden; [d]Department of Cardiology, Sahlgrenska University Hospital, Gothenburg, Sweden; [e]Health Economics & Outcomes Research, Amgen (Europe) GmbH, Rotkreuz, Switzerland; [f]Medical Affairs, Amgen AB, Stockholm, Sweden; [g]Sence Research, Uppsala, Sweden

Supplementary methods

Supplementary Methods 1. Characterisation of the study cohort

International Classification of Diseases (ICD) codes, Nordic Medico-Statistical Committee Classification of Surgical Procedures (NCSP) system procedure codes and Anatomical Therapeutic Chemical (ATC) classification system codes were used to identify clinical characteristics and lipid-lowering therapy(ies) (LLT) in the study cohort. Clinical characteristics could only be obtained from the National Patient Register from 1997–2019.

The following ICD-10/procedure codes recorded in any diagnosis position were used to identify patients with:

Atherosclerotic cardiovascular disease (ASCVD)

Coronary heart disease:

Description/procedure	Code
Acute myocardial infarction	I21
Angina pectoris	I20
Unstable angina	I20.0
Subsequent myocardial infarction	I22
Complications following acute myocardial infarction	I23
Other acute ischaemic heart diseases	I24
Chronic ischaemic heart disease	I25
Aorto-coronary venous bypass	FNC
Aorto-coronary bypass using prosthetic graft	FND
Coronary bypass using free arterial graft	FNE
Expansion and recanalisation of coronary artery	FNG

All stroke:

Description	Code
Subarachnoid haemorrhage	I60
Intracerebral haemorrhage	I61
Other non-traumatic intracranial haemorrhage	I62
Cerebral infarction	I63
Stroke, not specified as haemorrhage or infarction	I64
Transient cerebral ischaemic attacks and related syndromes	G45

Peripheral artery disease (PAD) (ICD-10 codes):

Description	Code
Atherosclerosis	I70
Aortic aneurysm and dissection	I71
Other aneurysm and dissection	I72
Thromboangiitis obliterans [Buerger]	I73.1
Peripheral vascular disease, unspecified	I73.9
Arterial embolism and thrombosis	I74
Arterial fibromuscular dysplasia	I77.3
Arteritis, unspecified	I77.6
Other specified disorders of arteries and arterioles	I77.8
Disorders of arteries, arterioles and capillaries in diseases classified elsewhere	I79

PAD (procedure codes):

Procedure	Code
Operations on arteries of aortic arch and branches	PA
Operations on arteries of upper extremity	PB
Operations on suprarenal abdominal aorta and visceral arteries	PC
Operations on infrarenal abdominal aorta and iliac arteries and distal connections	PD
Operations on femoral artery with branches and connection to popliteal artery	PE
Connection from femoral artery to infrapopliteal arteries and operations on popliteal artery and arteries of lower leg and foot	PF
Extra-anatomic bypass operations	PG

Peripheral (percutaneous transluminal angioplasty) revascularisation:

Procedure	Code
Thrombectomy or embolectomy of iliac artery	PDE30
Thrombendarteriectomy of iliac artery	PDF30
Bypass from infrarenal abdominal aorta and iliac arteries	PDH
Plastic repair of iliac artery	PDN30
Percutaneous plastic repair of iliac artery	PDP30
Insertion of stent into iliac artery	PDQ30
Thrombectomy or embolectomy of femoral artery and branches	PEE
Thrombendarteriectomy of femoral artery and branches	PEF
Bypass from femoral artery and branches	PEH
Plastic repair of femoral artery and branches	PEN
Percutaneous plastic repair of femoral artery and branches	PEP
Insertion of stent into femoral artery and branches	PEQ
Other operations on femoral artery with branches and connection to popliteal artery	PEW
Exploration of popliteal artery and arteries of lower leg and foot	PFA
Thrombectomy or embolectomy of popliteal artery and arteries of lower leg and foot	PFE
Bypass from femoral artery to infrapopliteal arteries and from popliteal artery to arteries of lower leg and foot	PFH

Plastic repair of popliteal artery	PFN
Percutaneous plastic repair of popliteal artery or artery of lower leg	PFP
Insertion of stent into popliteal artery or artery of lower leg	PFQ
Repair after previous bypass from femoral or popliteal artery to infrapopliteal arteries and reconstruction of popliteal artery and arteries of lower leg and foot	PFU
Other connections from femoral artery to infrapopliteal arteries and operations on popliteal artery and arteries of lower leg and foot	PFW
Extra-anatomic bypass	PGH
Repair of extra-anatomic bypass	PGU
Other extra-anatomic bypass operations	PGW
Percutaneous transluminal dilatation of leg artery	DP008
Percutaneous transluminal dilatation of iliac artery	DP010

Diseases of the circulatory system

Description	Code
Diseases of the circulatory system	I00-I99

Diabetes mellitus

Description	Code
Diabetes mellitus	E10-E14

Renal failure

Description	Code
Renal failure	N17-N19

Lipoprotein metabolism disorders/other lipidaemias

Description	Code
Lipoprotein metabolism disorders/other lipidaemias	E78
Lipoprotein apheresis	DR001
Familial hypercholesterolaemia (FH)	E78.0A

Identifying LLT

The following ATC codes were used to identify patients using LLT:

Drug	Code
Simvastatin	C10AA01
Lovastatin	C10AA02
Pravastatin	C10AA03
Fluvastatin	C10AA04
Atorvastatin	C10AA05
Cerivastatin	C10AA06
Rosuvastatin	C10AA07
Pitavastatin	C10AA08
Evolocumab	C10AX13
Ezetimibe	C10AX09
Lovastatin and nicotinic acid	C10BA01
Simvastatin and ezetimibe	C10BA02
Pravastatin and fenofibrate	C10BA03
Simvastatin and fenofibrate	C10BA04
Atorvastatin and ezetimibe	C10BA05
Rosuvastatin and ezetimibe	C10BA06
Rosuvastatin and omega-3 fatty acids	C10BA07
Atorvastatin and omega-3 fatty acids	C10BA08
Rosuvastatin and fenofibrate	C10BA09
Simvastatin and acetylsalicylic acid	C10BX01
Pravastatin and acetylsalicylic acid	C10BX02
Atorvastatin and amlodipine	C10BX03
Simvastatin, acetylsalicylic acid and ramipril	C10BX04
Rosuvastatin and acetylsalicylic acid	C10BX05
Atorvastatin, acetylsalicylic acid and ramipril	C10BX06
Rosuvastatin, amlodipine and lisinopril	C10BX07
Atorvastatin and acetylsalicylic acid	C10BX08
Rosuvastatin and amlodipine	C10BX09
Rosuvastatin and valsartan	C10BX10
Atorvastatin, amlodipine and perindopril	C10BX11
Atorvastatin, acetylsalicylic acid and perindopril	C10BX12
Rosuvastatin, perindopril and indapamide	C10BX13
Rosuvastatin, amlodipine and perindopril	C10BX14
Atorvastatin and perindopril	C10BX15
Rosuvastatin and fimasartan	C10BX16
Rosuvastatin and ramipril	C10BX17

Supplementary Methods 2. Identifying FH patients using the Dutch Lipid Clinic Network (DLCN) criteria

As the ICD-10 code for FH was only implemented into the Swedish ICD system on 1 January 2019, the highest low-density lipoprotein cholesterol (LDL-C) and total cholesterol concentrations on record were used to identify potential FH in each patient using the DLCN criteria (1) where data were available.

- Two points are marked to a patient if there is premature coronary heart disease and/or if there is premature cerebral or peripheral vascular disease (premature defined as <55 years for males and <60 years for females).
- The number of points that were marked to a patient for their highest LDL-C and total cholesterol concentrations on record were:
  - 8 points if LDL-C was ≥8.5 mmol/L, total cholesterol >10.25 mmol/L
  - 5 points if LDL-C was 6.5–8.4 mmol/L, total cholesterol 8.25–10.24 mmol/L
  - 3 points if LDL-C was 5.0–6.4 mmol/L, total cholesterol 6.75–8.24 mmol/L
  - 1 point if LDL-C was 4.0–4.9 mmol/L, total cholesterol 5.75–6.74 mmol/L.

A diagnosis of FH was made according to the number of points accumulated using the above steps:

- >8 points = definitive FH.
- 6–8 points = probable FH.
- 3–5 points = possible FH.
- 0–2 points = unlikely FH.

Supplementary Methods 3. Persistence with evolocumab

For the assessment of persistence with evolocumab, several sensitivity analyses were conducted to address other reasons for inconsistencies in medication use. The sensitivity analyses performed were: 1) First 28d, inpatient: if a patient was admitted to hospital due to cardiovascular disease, the number of days they spent in inpatient care (where they would not be using their own medications) was also added to the number of days covered by the relevant prescription, in addition to a permissible gap of 28 days; 2) First 28d, overlap: if a subsequent prescription for evolocumab was filled before the number of days covered by the previous prescription ended, then the number of days overlapping between that new prescription being filled and the end of the previous coverage period were added to the subsequent coverage period, in addition to a permissible gap of 28 days (accounting for any build-up in evolocumab supply); and 3) First 28d, 25% grace: coverage periods for all prescriptions, inclusive of a permissible gap of 28 days, were extended by an additional 25% grace period. Additionally, an analysis (First 28d and Last 28d) only allowing a permissible gap of 28 days was performed. As with the primary analysis, if a patient didn’t fill the next prescription before any of these periods of coverage ended, they were marked as non-persistent for that occasion.

For the primary and sensitivity analyses, a last incident of non-persistence was defined as those where there was no subsequent filled prescription for evolocumab. Patients were censored when deemed non-persistent or at death. To be included in these analyses, the follow-up period for a given patient needed to be at least 28 or 56 days, dependent on the analysis being conducted.

Supplementary Methods 4. LDL-C goal achievement

Regarding LDL-C goal achievement, a multi-state Markov model of panel data (2) was fitted with the transient LDL-C states ‘<1.4 mmol/L’, ‘≥1.4 to <1.8 mmol/L’, and ‘≥1.8 mmol/L’. Data were used as in the generalised least squares regression models, based on patients with complete follow-up for 180 days. Piecewise constant transition intensities were assumed between time cutoffs at –14, 0, 14, 28 and 90 days, over ±180 days around the time of evolocumab initiation. Models were adjusted for adherence categories and prior ASCVD history. Patient self-selection (2) was assumed to be rare in relation to LDL-C sampling.

Supplementary results

Supplementary Result 1. Persistence with evolocumab throughout 3 years of follow-up

Supplementary Table 1. Persistence with evolocumab in the overall cohort at different time-points during the first 3 years, from the date that treatment was first initiated.

	Proportion of cohort persistent at each time point				
	6 months	9 months	12 months	24 months	36 months
	n = 1,768	*n* = 1,459	*n* = 1,224	*n* = 555	*n* = 280
First 28d	0.70	0.59	0.49	0.31	0.22
First 56d	0.78	0.72	0.65	0.52	0.42
Last 28d	0.81	0.77	0.74	0.64	0.57
Last 56d	0.82	0.79	0.76	0.69	0.61

The proportion of the cohort persistent during each predefined treatment-period of interest, according to the refill-gap using four different gap definitions, are reported:

First 28d and Last 28d: sensitivity analysis where a permissible gap of 28 days was added to the number of days covered; First 56d and Last 56d: base case where a permissible gap of 56 days was added to the number of days covered.

Supplementary Result 2. Persistence with evolocumab in those with and without ASCVD during the first 12 months of follow-up

The number of days from the date evolocumab treatment was first initiated to the first and last incidents of non-persistence are presented for each patient, together with the proportion of patients deemed persistent with their evolocumab treatment. The seven lines represent the different gap definitions used in the analyses: First 56d and Last 56d: base case where a permissible gap of 56 days was added to the number of days covered; First 28d and Last 28d: sensitivity analysis where a permissible gap of 28 days was added to the number of days covered; First 28d, inpatient: sensitivity analysis where the number of days a patient spent in inpatient care for a cardiovascular disease admission was added (in addition to a permissible gap of 28 days) to the number of days covered; First 28d, overlap: sensitivity analysis where the number of overlapping days between a new evolocumab prescription being filled before the end of the previous coverage period were added (in addition to a permissible gap of 28 days) to the subsequent coverage period; First 28d, 25% grace period: sensitivity analysis where coverage periods inclusive of a permissible gap of 28 days were extended by an additional 25%. A substantial reduction in persistence is observable at Day 84 in all analyses due to the patients who discontinued evolocumab treatment after their first filled prescription.

Supplementary Figure 1. Kaplan–Meier curve of persistence with evolocumab in those with ASCVD during the first 12 months of follow-up.

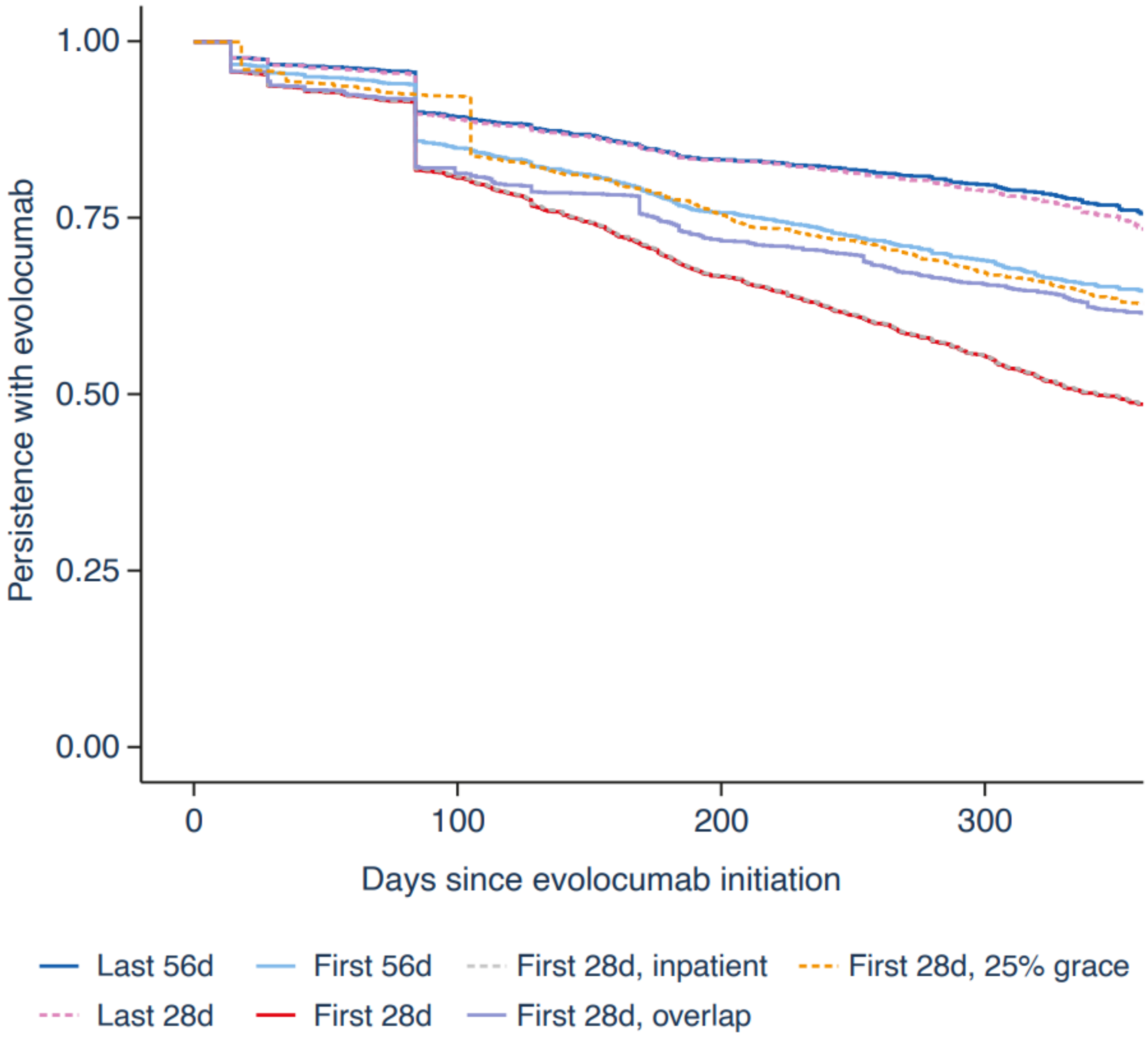

ASCVD: atherosclerotic cardiovascular disease.

Supplementary Figure 2. Kaplan–Meier curve of persistence with evolocumab in those without ASCVD during the first 12 months of follow-up.

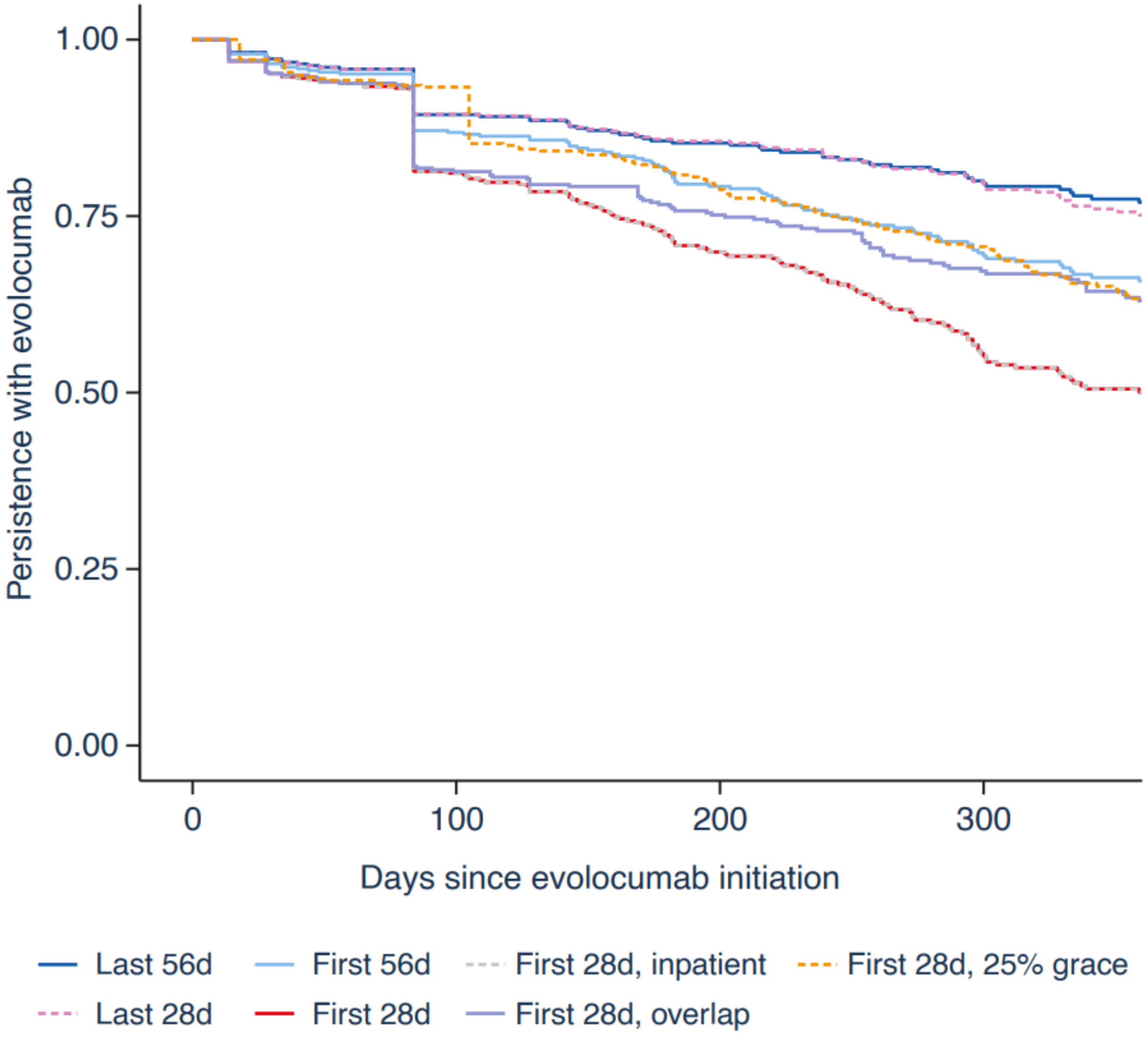

ASCVD: atherosclerotic cardiovascular disease.

Supplementary Result 3. Persistence with other LLT during the first 12 months of follow-up

Supplementary Figure 3. Kaplan–Meier curve of persistence with other LLT during the first 12 months of follow-up.

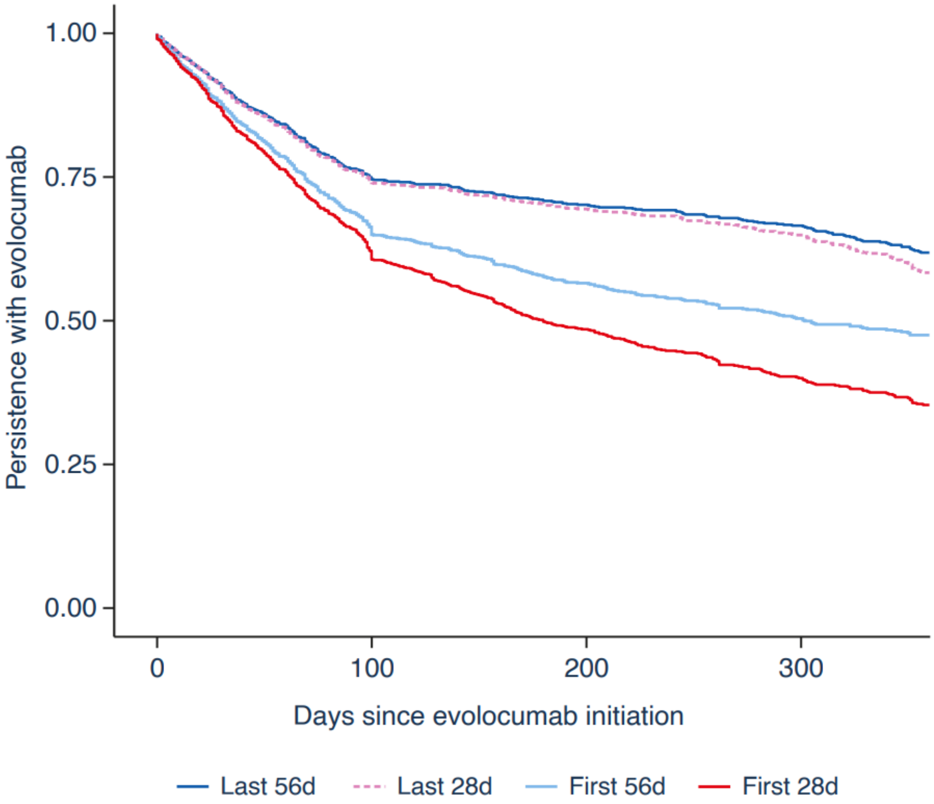

The number of days from the date evolocumab treatment was first initiated to the first and last incidents of non-persistence with another LLT are presented for each patient, together with the proportion of patients deemed persistent with their treatment. The four lines represent the different gap definitions used in the analyses: First 56d and Last 56d: base case where a permissible gap of 56 days was added to the number of days covered; First 28d and Last 28d: sensitivity analysis where a permissible gap of 28 days was added to the number of days covered.

LLT: lipid-lowering therapy(ies).

Supplementary Result 4. Adherence to evolocumab during the first 3 years of follow-up

Patients with a proportion of days covered of ≥0.8 for evolocumab were deemed adherent to treatment. Only patients with complete follow-up for each given follow-up period were included in the analyses. Four types of analyses were conducted: Base: the base case where a permissible gap of 56 days was added to the number of days covered; Inpatient: sensitivity analysis where the number of days a patient spent in inpatient care for a cardiovascular disease admission was added (in addition to a permissible gap of 28 days) to the number of days covered; 25% grace: sensitivity analysis where coverage periods inclusive of a permissible gap of 28 days were extended by an additional 25%; Overlap: sensitivity analysis where the number of overlapping days between a new evolocumab prescription being filled before the end of the previous coverage period were added (in addition to the permissible gap of 28 days) to the subsequent coverage period. The analyses that were adjusted for persistence only included those who did not record a discontinuation in evolocumab treatment (>56-day medication gap).

Supplementary Table 2. Adherence to evolocumab in the overall cohort during the first 3 years of follow-up.

		Not adjusted for persistence				Adjusted for persistence			
**Analysis**	**Months**	**n**	**PDC, mean (SD)**	**PDC, median (IQR)**	**Proportion with PDC ≥0.8**	**n**	**PDC, mean (SD)**	**PDC, median (IQR)**	**Proportion with PDC ≥0.8**
Base	6	1,768	0.85 (0.22)	0.95 (0.82–1.00)	0.77	1,462	0.93 (0.12)	0.97 (0.91–1.00)	0.90
Base	9	1,459	0.81 (0.25)	0.93 (0.75–0.98)	0.72	1,167	0.91 (0.13)	0.95 (0.89–0.99)	0.87
Base	12	1,224	0.77 (0.27)	0.90 (0.70–0.96)	0.69	941	0.89 (0.13)	0.93 (0.86–0.97)	0.86
Base	24	555	0.70 (0.31)	0.86 (0.50–0.94)	0.60	400	0.86 (0.15)	0.91 (0.83–0.95)	0.81
Base	36	280	0.65 (0.33)	0.82 (0.38–0.92)	0.53	184	0.85 (0.15)	0.90 (0.82–0.94)	0.79
Inpatient	6	1,768	0.85 (0.22)	0.95 (0.83–1.00)	0.77	1,465	0.93 (0.12)	0.97 (0.91–1.00)	0.89
Inpatient	9	1,459	0.81 (0.25)	0.93 (0.75–0.98)	0.72	1,168	0.91 (0.13)	0.95 (0.89–0.99)	0.87
Inpatient	12	1,224	0.78 (0.27)	0.90 (0.70–0.96)	0.69	943	0.89 (0.13)	0.93 (0.86–0.97)	0.86
Inpatient	24	555	0.70 (0.31)	0.86 (0.50–0.94)	0.60	400	0.86 (0.15)	0.91 (0.83–0.95)	0.81
Inpatient	36	280	0.65 (0.33)	0.82 (0.38–0.92)	0.53	184	0.85 (0.15)	0.90 (0.82–0.94)	0.79
25% grace	6	1,768	0.90 (0.21)	1.00 (0.93–1.00)	0.83	1,503	0.97 (0.10)	1.00 (1.00–1.00)	0.94
25% grace	9	1,459	0.86 (0.24)	1.00 (0.85–1.00)	0.77	1,190	0.95 (0.11)	1.00 (0.97–1.00)	0.93
25% grace	12	1,224	0.83 (0.26)	0.98 (0.79–1.00)	0.75	960	0.94 (0.12)	1.00 (0.94–1.00)	0.92
25% grace	24	555	0.76 (0.32)	0.94 (0.59–1.00)	0.65	406	0.92 (0.14)	0.98 (0.91–1.00)	0.87
25% grace	36	280	0.71 (0.34)	0.91 (0.42–0.99)	0.60	186	0.91 (0.14)	0.97 (0.90–1.00)	0.86

Overlap	6	1,768	0.88 (0.23)	1.00 (0.91–1.00)	0.81	1,485	0.95 (0.12)	1.00 (0.97–1.00)	0.93
Overlap	9	1,459	0.85 (0.26)	1.00 (0.83–1.00)	0.76	1,179	0.95 (0.13)	1.00 (0.96–1.00)	0.92
Overlap	12	1,224	0.82 (0.28)	0.99 (0.75–1.00)	0.73	954	0.94 (0.13)	1.00 (0.94–1.00)	0.91
Overlap	24	555	0.76 (0.33)	0.95 (0.58–1.00)	0.65	406	0.92 (0.15)	1.00 (0.92–1.00)	0.87
Overlap	36	280	0.71 (0.35)	0.92 (0.40–1.00)	0.59	187	0.92 (0.15)	0.99 (0.91–1.00)	0.85

IQR: interquartile range; PDC: proportion of days covered; SD: standard deviation.

Supplementary Table 3. Adherence to evolocumab in those with ASCVD during the first 3 years of follow-up.

		Not adjusted for persistence				Adjusted for persistence			
**Analysis**	**Months**	**n**	**PDC, mean (SD)**	**PDC, median (IQR)**	**Proportion with PDC ≥0.8**	**n**	**PDC, mean (SD)**	**PDC, median (IQR)**	**Proportion with PDC ≥0.8**
Base	6	1,406	0.85 (0.23)	0.95 (0.82–1.00)	0.77	1,156	0.92 (0.13)	0.97 (0.91–1.00)	0.90
Base	9	1,159	0.81 (0.25)	0.92 (0.75–0.98)	0.72	923	0.90 (0.13)	0.95 (0.88–0.99)	0.87
Base	12	973	0.77 (0.27)	0.90 (0.70–0.96)	0.69	749	0.89 (0.13)	0.93 (0.86–0.97)	0.86
Base	24	432	0.70 (0.31)	0.87 (0.47–0.94)	0.60	307	0.87 (0.14)	0.91 (0.84–0.95)	0.82
Base	36	219	0.64 (0.34)	0.83 (0.29–0.92)	0.54	142	0.85 (0.15)	0.91 (0.83–0.94)	0.82
Inpatient	6	1,406	0.85 (0.23)	0.95 (0.82–1.00)	0.77	1,159	0.92 (0.13)	0.97 (0.91–1.00)	0.89
Inpatient	9	1,159	0.81 (0.25)	0.92 (0.75–0.98)	0.72	924	0.90 (0.13)	0.95 (0.89–0.99)	0.87
Inpatient	12	973	0.78 (0.27)	0.90 (0.70–0.96)	0.69	751	0.89 (0.13)	0.93 (0.86–0.97)	0.86
Inpatient	24	432	0.70 (0.31)	0.87 (0.47–0.94)	0.60	307	0.87 (0.14)	0.92 (0.84–0.95)	0.82
Inpatient	36	219	0.65 (0.34)	0.83 (0.29–0.92)	0.54	142	0.86 (0.14)	0.91 (0.83–0.95)	0.82
25% grace	6	1,406	0.90 (0.21)	1.00 (0.92–1.00)	0.82	1,187	0.96 (0.10)	1.00 (1.00–1.00)	0.94
25% grace	9	1,159	0.86 (0.24)	1.00 (0.85–1.00)	0.77	941	0.95 (0.11)	1.00 (0.97–1.00)	0.93
25% grace	12	973	0.83 (0.26)	0.98 (0.79–1.00)	0.75	766	0.94 (0.12)	1.00 (0.95–1.00)	0.92
25% grace	24	432	0.76 (0.32)	0.94 (0.56–1.00)	0.65	313	0.92 (0.14)	0.98 (0.92–1.00)	0.88
25% grace	36	219	0.70 (0.36)	0.92 (0.32–0.99)	0.58	142	0.92 (0.14)	0.97 (0.92–1.00)	0.85

Overlap	6	1,406	0.88 (0.23)	1.00 (0.91–1.00)	0.81	1,173	0.95 (0.12)	1.00 (0.97–1.00)	0.93
Overlap	9	1,159	0.85 (0.26)	1.00 (0.82–1.00)	0.76	934	0.95 (0.13)	1.00 (0.96–1.00)	0.91
Overlap	12	973	0.82 (0.28)	0.99 (0.76–1.00)	0.73	761	0.94 (0.13)	1.00 (0.94–1.00)	0.90
Overlap	24	432	0.76 (0.33)	0.95 (0.51–1.00)	0.66	312	0.93 (0.15)	1.00 (0.93–1.00)	0.89
Overlap	36	219	0.70 (0.37)	0.92 (0.32–1.00)	0.60	145	0.93 (0.14)	0.99 (0.92–1.00)	0.87

ASCVD: atherosclerotic cardiovascular disease; IQR: interquartile range; PDC: proportion of days covered; SD: standard deviation.

Supplementary Table 4. Adherence to evolocumab in those without ASCVD during the first 3 years of follow-up.

			Not adjusted for persistence				Adjusted for persistence		
**Analysis**	**Months**	**n**	**PDC, mean (SD)**	**PDC, median (IQR)**	**Proportion with PDC ≥0.8**	**n**	**PDC, mean (SD)**	**PDC, median (IQR)**	**Proportion with PDC ≥0.8**
Base	6	362	0.86 (0.22)	0.96 (0.85–1.00)	0.79	306	0.93 (0.11)	0.98 (0.91–1.00)	0.90
Base	9	300	0.82 (0.25)	0.93 (0.76–0.98)	0.73	244	0.91 (0.13)	0.94 (0.89–0.99)	0.87
Base	12	251	0.77 (0.27)	0.89 (0.70–0.96)	0.66	192	0.89 (0.13)	0.92 (0.86–0.98)	0.83
Base	24	123	0.71 (0.29)	0.82 (0.58–0.92)	0.58	93	0.84 (0.16)	0.89 (0.80–0.94)	0.76
Base	36	61	0.69 (0.29)	0.80 (0.55–0.90)	0.49	42	0.83 (0.15)	0.87 (0.79–0.91)	0.71
Inpatient	6	362	0.86 (0.22)	0.96 (0.85–1.00)	0.79	306	0.93 (0.11)	0.98 (0.91–1.00)	0.90
Inpatient	9	300	0.82 (0.25)	0.93 (0.76–0.98)	0.73	244	0.91 (0.13)	0.94 (0.89–0.99)	0.87
Inpatient	12	251	0.77 (0.27)	0.89 (0.70–0.96)	0.66	192	0.89 (0.13)	0.92 (0.86–0.98)	0.83
Inpatient	24	123	0.71 (0.29)	0.82 (0.58–0.92)	0.58	93	0.84 (0.16)	0.89 (0.80–0.94)	0.76
Inpatient	36	61	0.69 (0.29)	0.80 (0.55–0.90)	0.49	42	0.83 (0.15)	0.87 (0.79–0.91)	0.71
25% grace	6	362	0.91 (0.20)	1.00 (0.96–1.00)	0.85	316	0.97 (0.08)	1.00 (1.00–1.00)	0.95
25% grace	9	300	0.87 (0.24)	1.00 (0.86–1.00)	0.78	249	0.96 (0.11)	1.00 (0.97–1.00)	0.92
25% grace	12	251	0.83 (0.26)	0.97 (0.79–1.00)	0.75	194	0.94 (0.11)	1.00 (0.94–1.00)	0.93
25% grace	24	123	0.77 (0.30)	0.91 (0.68–0.99)	0.64	93	0.91 (0.15)	0.96 (0.88–1.00)	0.84
25% grace	36	61	0.75 (0.30)	0.90 (0.66–0.95)	0.64	44	0.90 (0.14)	0.95 (0.89–0.98)	0.89

Overlap	6	362	0.89 (0.22)	1.00 (0.93–1.00)	0.82	312	0.96 (0.10)	1.00 (0.97–1.00)	0.92
Overlap	9	300	0.86 (0.25)	1.00 (0.84–1.00)	0.77	245	0.95 (0.12)	1.00 (0.95–1.00)	0.92
Overlap	12	251	0.82 (0.28)	0.98 (0.76–1.00)	0.73	193	0.94 (0.13)	1.00 (0.94–1.00)	0.91
Overlap	24	123	0.77 (0.31)	0.94 (0.62–1.00)	0.63	94	0.91 (0.16)	0.99 (0.88–1.00)	0.80
Overlap	36	61	0.74 (0.31)	0.89 (0.55–1.00)	0.57	42	0.89 (0.17)	0.99 (0.87–1.00)	0.79

ASCVD: atherosclerotic cardiovascular disease; IQR: interquartile range; PDC: proportion of days covered; SD: standard deviation.

Supplementary Result 5. Changes in LDL-C following initiation of evolocumab treatment

The upper horizontal dashed line denotes the pre-evolocumab treatment mean LDL-C level, and the lower dashed line denotes the post-evolocumab treatment mean LDL-C level. The dashed vertical lines represent the day before treatment was initiated at day 0, and the post-evolocumab treatment measurement 90 days after initiation.

Supplementary Figure 4. Change in LDL-C level prior to evolocumab treatment initiation to 90 days after treatment in the overall cohort of 724 patients who recorded measurements of LDL-C levels during the 180 days before and the 180 days after evolocumab treatment was initiated. In this specific analysis, mean levels of LDL-C reduced by approximately 48% after evolocumab treatment was first initiated.

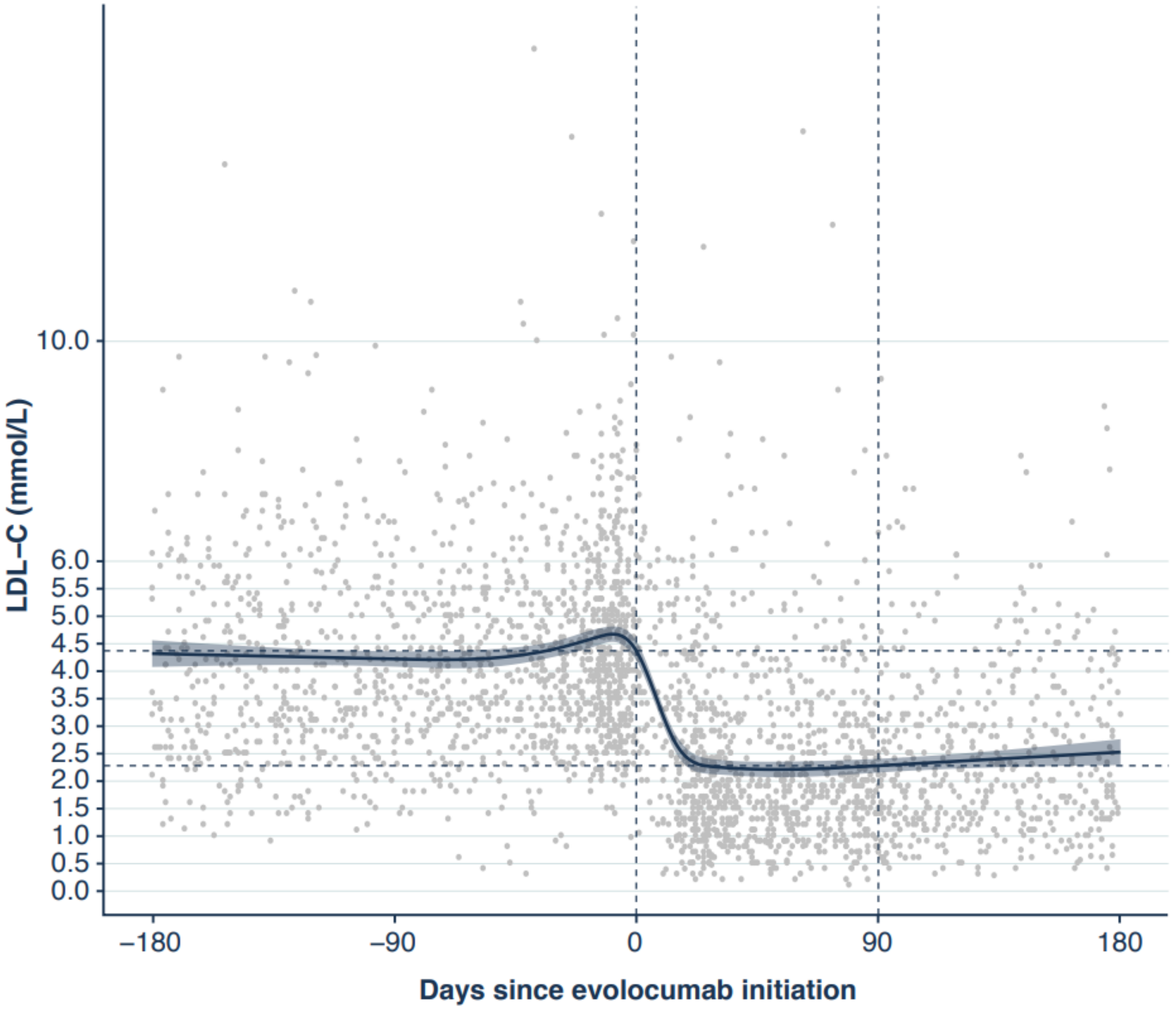

LDL-C: low-density lipoprotein cholesterol.

Supplementary Figure 5. Change in LDL-C level prior to evolocumab treatment initiation to 90 days after treatment in the overall cohort of 567 patients who were adherent to evolocumab treatment; and who had recorded measurements of LDL-C levels during the 180 days before and the 180 days after evolocumab treatment was initiated. In this specific analysis, mean levels of LDL-C reduced by approximately 53% after evolocumab treatment was first initiated.

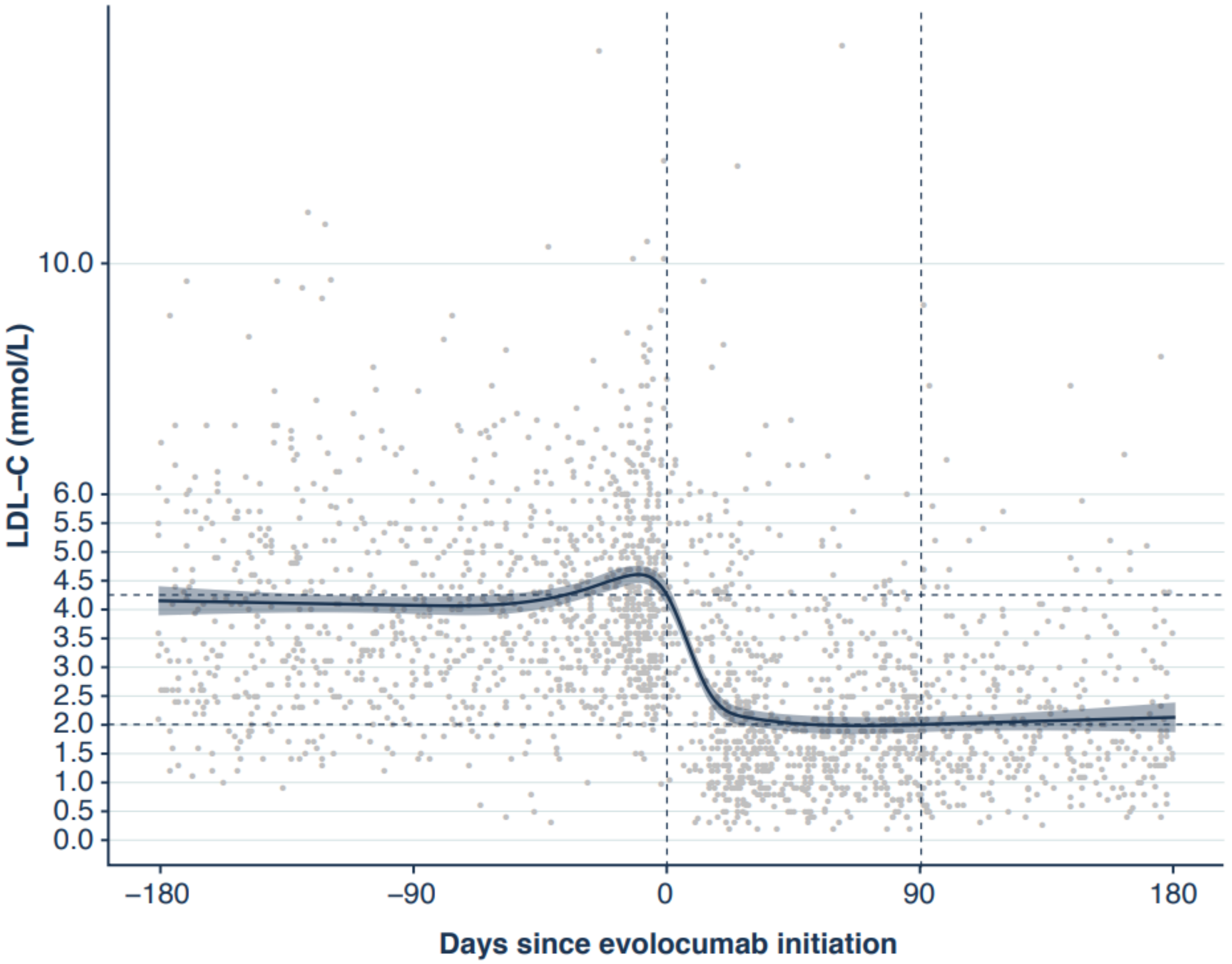

LDL-C: low-density lipoprotein cholesterol.

Supplementary Figure 6. Change in LDL-C level prior to evolocumab treatment initiation to 90 days after treatment in the overall cohort of 186 patients who were adherent to evolocumab treatment and oral LLT; and who had recorded measurements of LDL-C levels during the 180 days before and the 180 days after evolocumab treatment was initiated. In this specific analysis, mean levels of LDL-C reduced by approximately 59% after evolocumab treatment was first initiated.

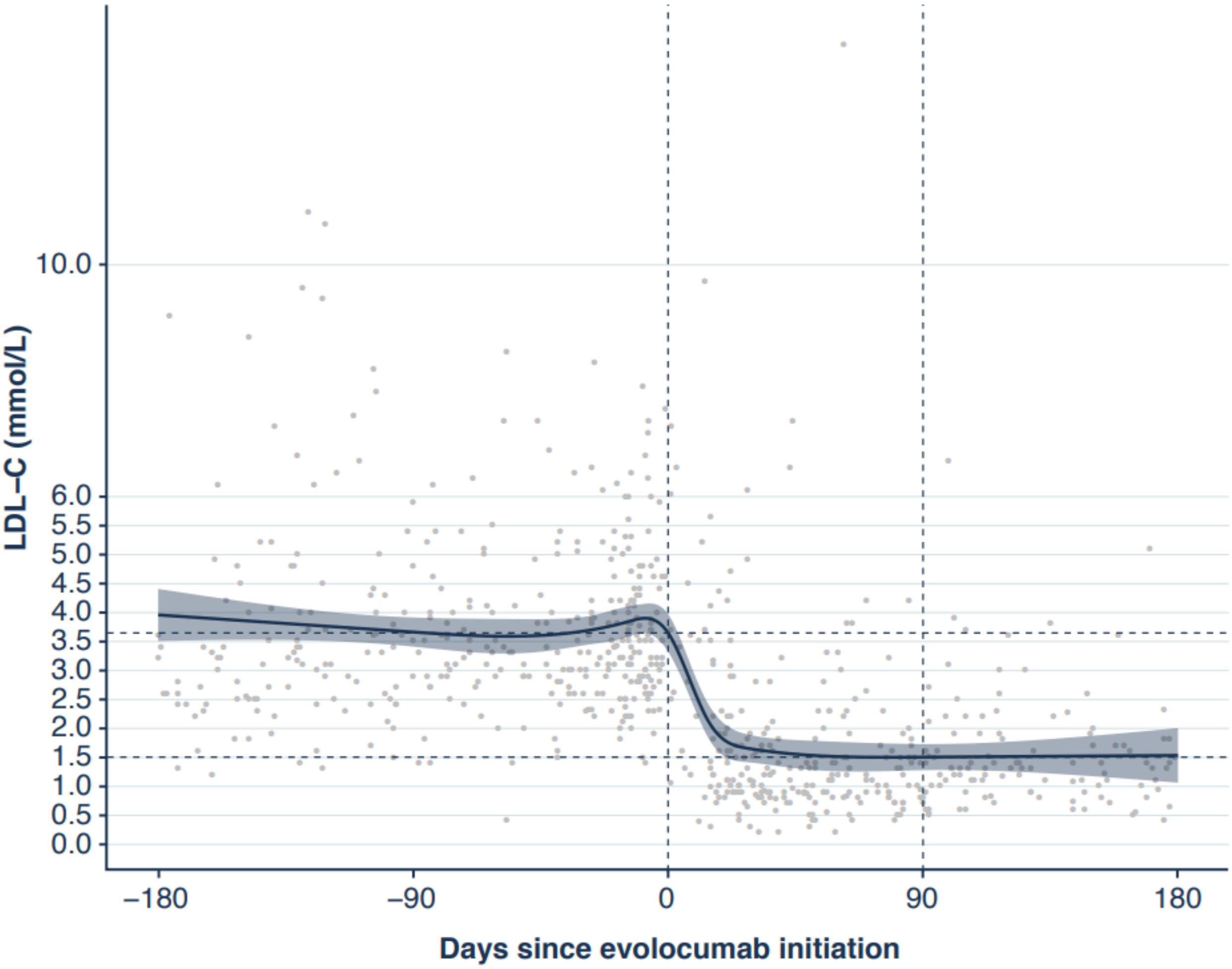

LDL-C: low-density lipoprotein cholesterol; LLT: lipid-lowering therapy(ies).

Supplementary Figure 7. Change in LDL-C level prior to evolocumab treatment initiation to 90 days after treatment in 571 patients with ASCVD who recorded measurements of LDL-C levels during the 180 days before and the 180 days after evolocumab treatment was initiated. In this specific analysis, mean levels of LDL-C reduced by approximately 50% after evolocumab treatment was first initiated.

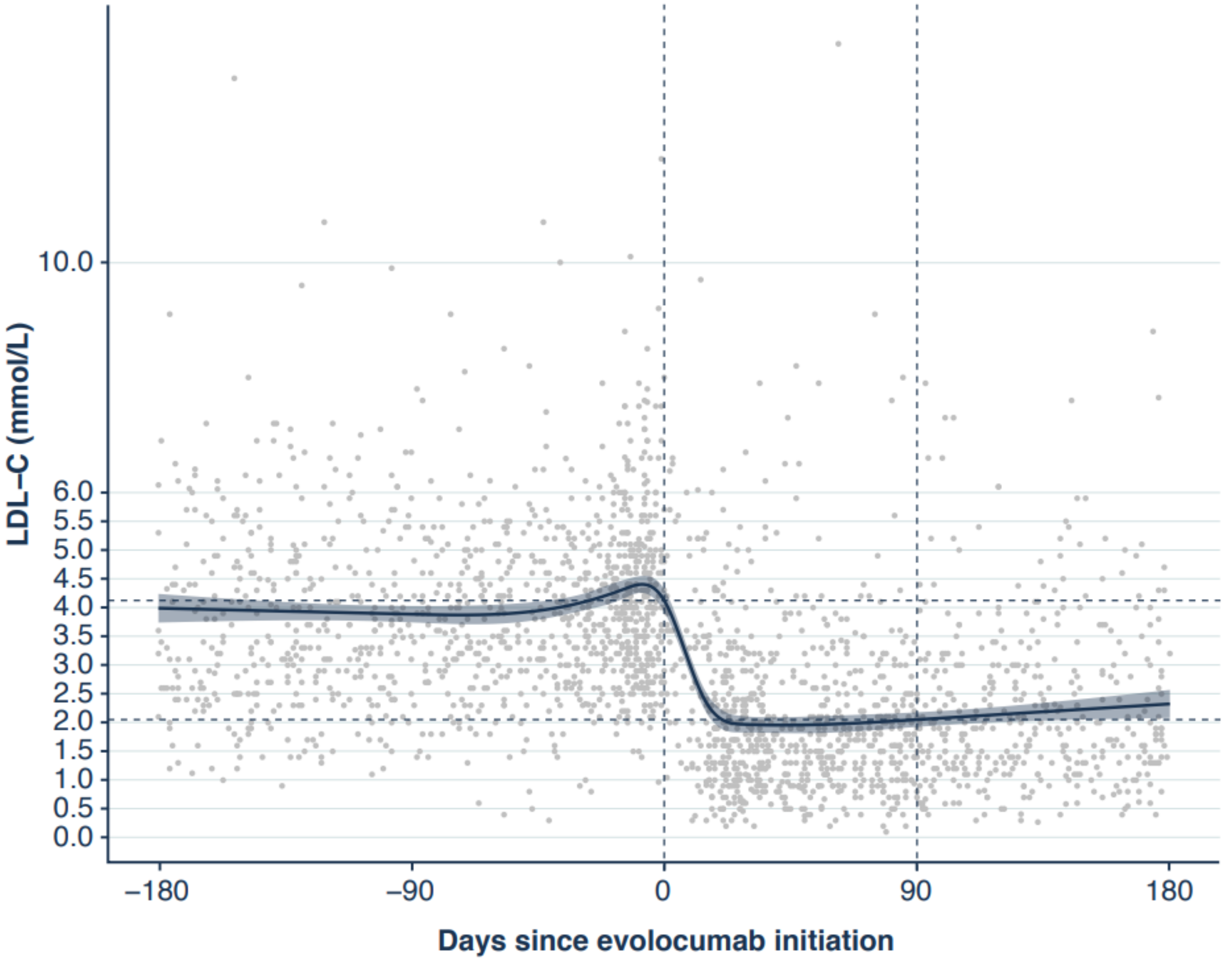

ASCVD: atherosclerotic cardiovascular disease; LDL-C: low-density lipoprotein cholesterol.

Supplementary Figure 8. Change in LDL-C level prior to evolocumab treatment initiation to 90 days after treatment in 447 patients with ASCVD who were adherent to evolocumab treatment; and who recorded measurements of LDL-C levels during the 180 days before and the 180 days after evolocumab treatment was initiated. In this specific analysis, mean levels of LDL-C reduced by approximately 55% after evolocumab treatment was first initiated.

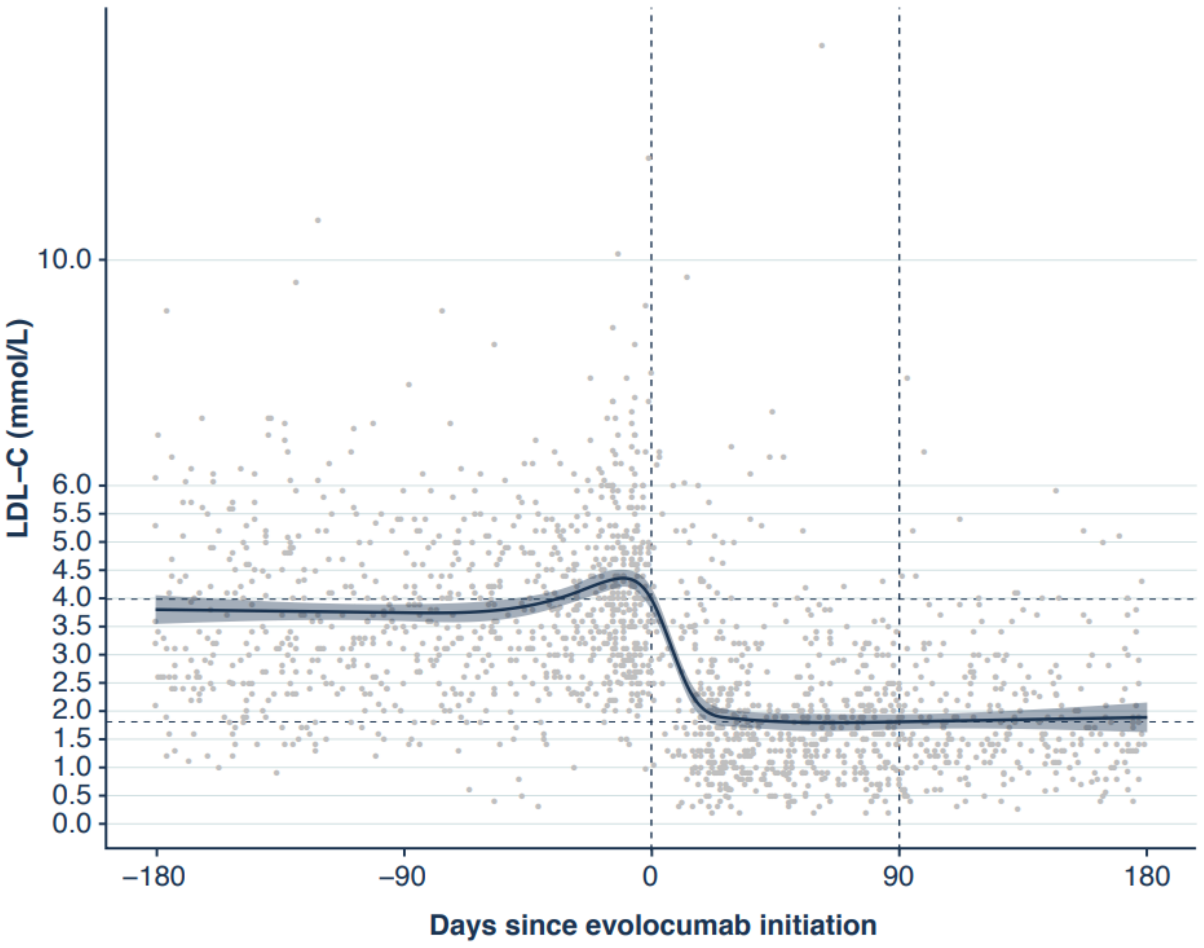

ASCVD: atherosclerotic cardiovascular disease; LDL-C: low-density lipoprotein cholesterol.

Supplementary Figure 9. Change in LDL-C level prior to evolocumab treatment initiation to 90 days after treatment in 152 patients with ASCVD who were adherent to evolocumab treatment and oral LLT; and who recorded measurements of LDL-C levels during the 180 days before and the 180 days after evolocumab treatment was initiated. In this specific analysis, mean levels of LDL-C reduced by approximately 58% after evolocumab treatment was first initiated.

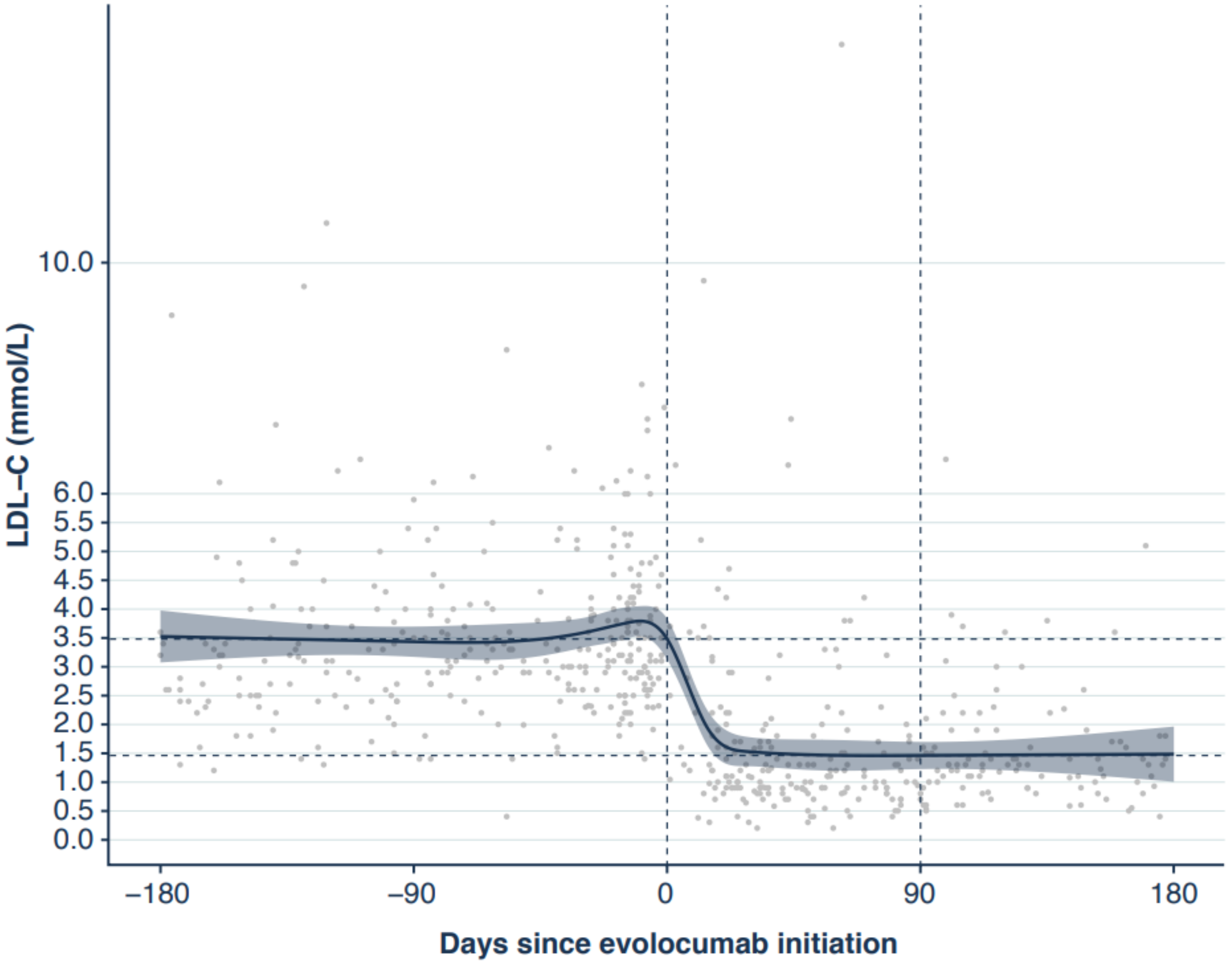

ASCVD: atherosclerotic cardiovascular disease; LDL-C: low-density lipoprotein cholesterol; LLT: lipid-lowering therapy(ies).

Supplementary Figure 10. Change in LDL-C level prior to evolocumab treatment initiation to 90 days after treatment in 153 patients without ASCVD who recorded measurements of LDL-C levels during the 180 days before and the 180 days after evolocumab treatment was initiated. In this specific analysis, mean levels of LDL-C reduced by approximately 41% after evolocumab treatment was first initiated.

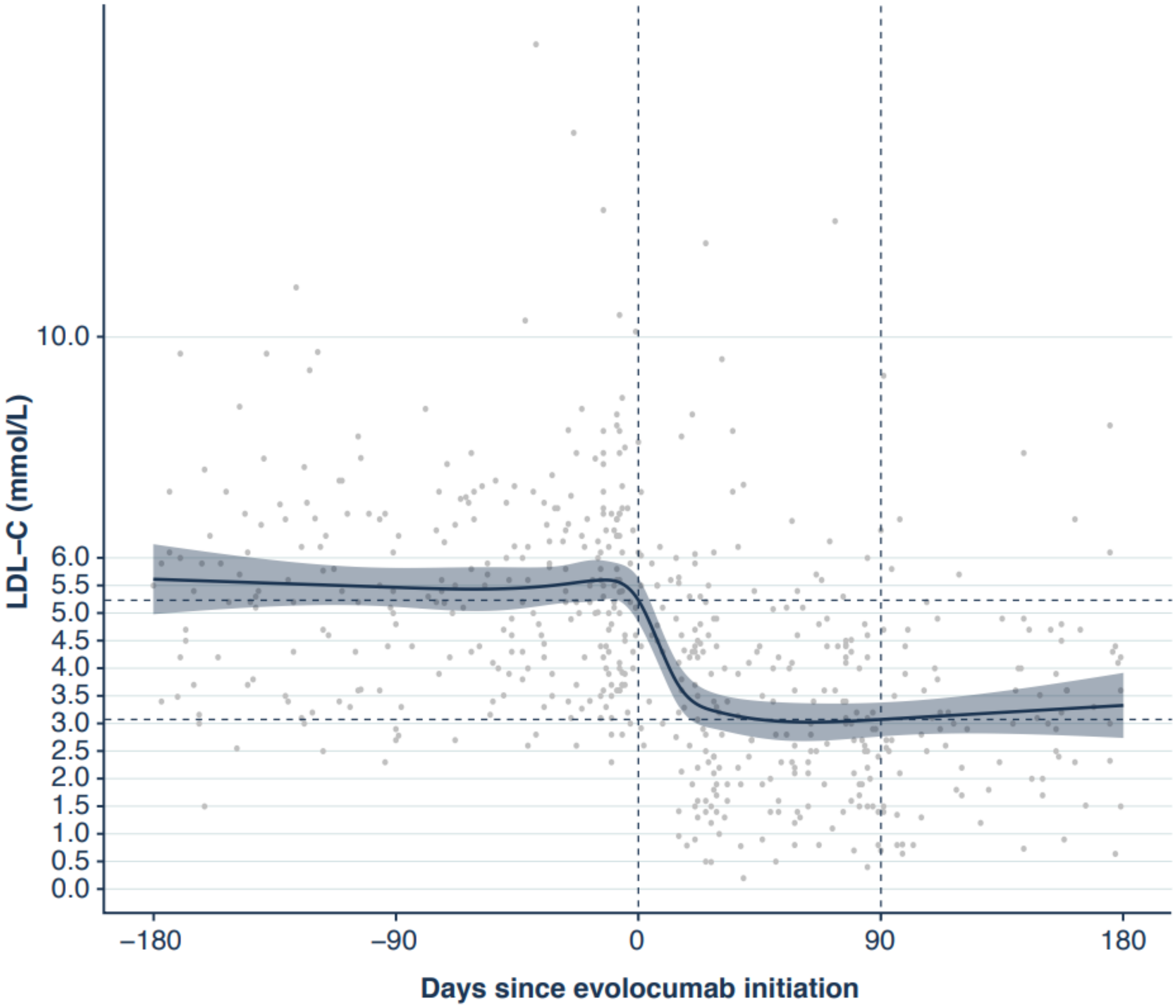

ASCVD: atherosclerotic cardiovascular disease; LDL-C: low-density lipoprotein cholesterol.

Supplementary Figure 11. Change in LDL-C level prior to evolocumab treatment initiation to 90 days after treatment in 120 patients without ASCVD who were adherent to evolocumab treatment; and who recorded measurements of LDL-C levels during the 180 days before and the 180 days after evolocumab treatment was initiated. In this specific analysis, mean levels of LDL-C reduced by approximately 47% after evolocumab treatment was first initiated.

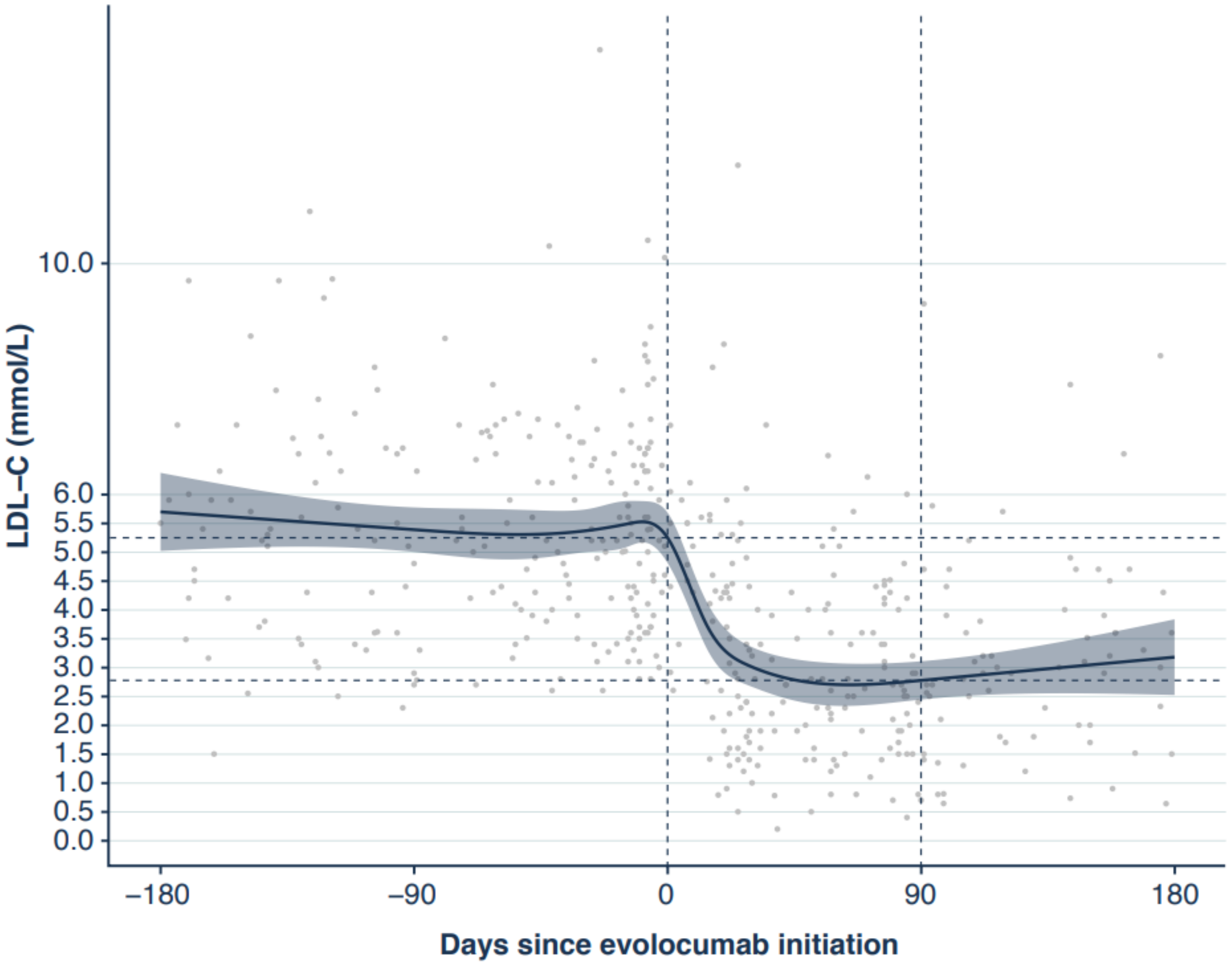

ASCVD: atherosclerotic cardiovascular disease; LDL-C: low-density lipoprotein cholesterol.

REFERENCES

1. Civeira F, International Panel on Management of Familial Hypercholesterolemia. Guidelines for the diagnosis and management of heterozygous familial hypercholesterolemia. Atherosclerosis. 2004;173:55-68. doi: 10.1016/j.atherosclerosis.2003.11.010.

2. Jackson C. Multi-State Models for Panel Data: The msm Package for R. J Stat Softw. 2011;38:1-28. doi: 10.18637/jss.v038.i08.
